# Leciplex Nanocarriers: An Optimized Platform for Thymol Delivery in Acne Management

**DOI:** 10.3390/pharmaceutics18070795

**Published:** 2026-06-28

**Authors:** Soha Elsalhy, Norhan Tantawy, Eman E. El Naggar, Wesam E. Gawad, Amira M. Badr, Reem T. Atawia, Jihad Mahmoud Alsofany

**Affiliations:** 1 Department of Pharmaceutics, College of Pharmaceutical Sciences and Drug Manufacturing, Misr University for Science and Technology (MUST), Giza 12585, Egypt; soha.mohamed@must.edu.eg; 2 Department of Pharmaceutics and Industrial Pharmacy, Faculty of Pharmacy, Capital University, Formerly Helwan University, Cairo P.O. Box 11795, Egypt; norhan.eltantawy@pharm.helwan.edu.eg; 3 Department of Pharmaceutical Technology, Faculty of Pharmacy, Horus University, New Damietta 34518, Egypt; eelnaggar@horus.edu.eg; 4 Department of Pharmaceutics, Faculty of Pharmacy, Mansoura National University, Gamasa 7731168, Egypt; 5 Department of Microbiology and Immunology, College of Pharmaceutical Sciences and Drug Manufacturing, Misr University for Science and Technology, 6th of October City 12566, Egypt; wesam.gawad@must.edu.eg; 6 Department of Pharmacology and Toxicology, College of Pharmacy, King Saud University, P.O. Box 2457, Riyadh 11415, Saudi Arabia; 7 Department of Pharmaceutical Sciences, College of Pharmacy, Southwestern Oklahoma State University, Weatherford, OK 73096, USA; reem.atawia@swosu.edu; 8 Department of Pharmacology and Toxicology, Faculty of Pharmacy, Ain Shams University, Cairo 11566, Egypt; 9 Department of Pharmaceutics, Faculty of Pharmacy, University of Sadat City, Sadat City 32897, Menoufia, Egypt

**Keywords:** Leciplex, thymol, nano-carrier, acne vulagris, I-optimal design

## Abstract

**Background/Objectives:** Antibiotics are commonly used for acne treatment. However, increasing bacterial resistance has prompted interest in natural antimicrobial agents, such as thymol (THY), as alternative therapies. This study investigated the effectiveness of Leciplex cationic nanovesicles encapsulating thymol (LPX-THY) as a promising topical acne management strategy. **Methods:** Leciplex nanovesicles were assembled using soy phosphatidylcholine (SPC) and cationic surfactants and characterized in terms of particle size, zeta potential, entrapment efficiency, morphology, in vitro release, and ex vivo skin permeation. The optimized formulation was subsequently incorporated into Carbopol/HPMC gel base and evaluated in terms of viscosity, in vitro release, ex vivo skin permeation, in vitro antimicrobial study, and in vivo assessment in a rat model. **Results:** Optimal THY-LPX nanovesicles made of SPC and Dimethyldidodecylammonium bromide DDAB in a 1:1 molar ratio showed circular outline with particle size, zeta potential, and entrapment efficiency of 187.7 ± 1.78 nm, 36.97 ±0.21 mV, and 60.5 ± 2.3%, respectively. THY-LPX gel demonstrated minimum inhibitory concentration (MIC) and minimum bactericidal concentration (MBC) at a concentration of 156.25 µg·mL^−1^ against Staphylococcus aureus, a clear absence of biofilm coating under SEM, and substantial red fluorescence, indicating reduction in viable bacteria under a confocal laser microscope. In vivo study showed enhanced anti-inflammatory effect evidenced by substantial ear skin thickness reduction; 72.7% for THY-LPX gel-treated rats compared to 41.7% and 20% for THY gel and blank LPX gel-treated groups, respectively. Histopathological investigation further confirmed reduced inflammatory response in rats treated with optimized THY-LPX gel. **Conclusions:** The developed THY-LPX gel serves as a potential topical delivery platform of THY for acne therapy.

## 1. Introduction

Acne vulgaris is a common chronic inflammatory condition caused by blocked follicles, excessive sebum, and bacteria, leading to pimples and cysts [1]. It is a multifactorial disease involving hair follicles and sebaceous glands, characterized by sebum buildup, bacterial growth, and inflammation. Acne ranges from comedones (blackheads/whiteheads) to inflammatory papules, pustules, and deep nodules [2]. Acne is caused by hormonal changes (androgens), excess oil production, and bacteria.

Human skin microbiota is generally benign. The two main genera of the typical skin microbiota are propionibacteria and staphylococci. Propionibacteria are primarily found inside sebaceous follicles, while staphylococci often colonize the skin’s surface. An imbalance between these bacterial populations can raise the risk of skin infections and conditions like acne [3]. Particularly important in the pathophysiology of acne are *Staphylococcus epidermidis* and *Staphylococcus aureus* (*S. aureus*), which are frequently found in affected sebaceous follicles [4]. Acne vulgaris and other skin infections are largely caused by the anaerobic bacteria *Propionibacterium acne* and *Staphylococcus aureus* colonizing the skin [4,5].

While *Cutibacterium acnes* is primarily responsible for acne vulgaris, *Staphylococcus aureus* frequently colonizes inflamed acne lesions, contributing to inflammation severity and increased antibiotic resistance due to long-term treatment [6].

*S. aureus*, a Gram-positive coccus, is a normally harmless bacterium of human skin and a principal pathogen in human suppurative infections. It may lead to various community and hospital infections, such as skin and soft tissue infections, pneumonia, and endocarditis [7]. Dreno et al. showed that *S. aureus* is more prevalent on the surfaces of pimples, papules, and pustules in acne sufferers, and its abundance positively correlates with acne severity, in contrast to unaffected skin [4].

Conventional acne treatments such as benzoyl peroxide and salicylic acid, in addition to topical antibiotics for *S. aureus* such as fusidic acid and mupirocin, are facing challenges due to increased drug resistance and adverse effects [8]. Alternative therapies are now necessary as a result. Natural products, particularly thymol (THY), are becoming popular due to their high efficacy, superior skin tolerability, antibacterial activity while preserving the healthy skin microbiome, fewer side effects, and the ability to avoid antibiotic resistance [9].

Thymol (THY) is a monoterpenoid phenolic compound derived from the thyme herb and correlated Lamiaceae species, has well-recognized broad-spectrum antibacterial and antibiofilm activity [10]. Former studies revealed its bacterial growth and biofilm inhibition potential [11,12]. Moreover, checkerboard/time-kill studies often demonstrate synergistic or additive interactions when THY is combined with conventional antibiotics [13]. This outstanding antimicrobial performance can reduce antibiotic minimum inhibitory concentrations (MICs) and enhance bactericidal activity, which in turn overcomes biofilm-associated tolerance. The direct therapeutic use of thymol is hindered by unfavorable physicochemical characteristics, including low water solubility, high volatility, and instability, which may contribute to poor bioavailability and increased toxicity at higher doses [14,15]. To address these limitations, the incorporation of thymol into nanoscale delivery systems has attracted considerable research interest. Previous studies have addressed the effectiveness of different nanocarriers in the topical delivery of THY for various skin disorders, including acne vulgaris [16,17,18]. Additionally, THY is a relatively non-toxic compound [19] and a previous in vivo 28-day oral toxicity study revealed that the no-observed effect level (NOAEL) of the essential oil of *Thymus vulgaris* was more than 250 mg/kg/day [20].

Leciplex (LPX), lecithin-based novel cationic nanocarriers, is a lipid nanovesicular system designed to enhance bioavailability. They are composed primarily of lecithin phosphatidylcholine and cationic surfactants, forming self-assembled, positively charged, vesicular structures. LPX systems are developed to improve the bioavailability of hydrophobic (water-insoluble) drugs using cationic surfactants such as CTAB (Cetyltrimethylammonium bromide) and DDAB (Didodecyldimethylammonium bromide). Several studies reported successful applications of LPX nanocarriers in drug delivery, especially for topical and transdermal delivery [21,22].

In the present study, we hypothesize that encapsulation of THY into a nanocarrier offers a promising platform for acne management with a safe, natural compound by localized and controlled THY release at the acne spots, thereby improving lesion exposure while reducing local irritation and systemic toxicity usually associated with conventional antibiotics and antimicrobials. Lipid-based nanocarriers, especially LPX, facilitate drug permeation across bacterial biofilms and disruption of the extracellular polymeric substance, promoting THY access to targeted bacterial cells [23,24]. In addition, nanoencapsulation protects THY from evaporation (volatile compound), inactivation, or dilution in pus and exudates of acne pustules [25]. Collectively, preclinical evidence indicates that nanoscale thymol-based formulations can enhance antimicrobial effect [26]. To the best of the authors’ knowledge, this is the first work to encapsulate thymol into LPX nanovesicles for enhanced topical skin delivery.

In the current study, we designed a topical gel matrix made of Carbopol and HPMC and loaded with LPX nanocarrier for effective topical delivery of THY. Furthermore, wide microbiological testing was performed on the *S. aureus* strain employing MIC testing of THY either free or loaded into LPX nanovesicle gel in addition to antibiofilm assay and structural visualization via electron microscope and confocal laser microscope imaging. In vivo assessment was accomplished in an acne-relevant model of *S. aureus*-infected rat skin after treatment with optimized gel formulation compared to THY, blank gel, as well as positive and negative control with inflammatory signs assessment and subsequent histopathological examination.

## 2. Materials and Methods

### 2.1. Materials

Thymol (98.5%) CAS. No.89-83-8; Sigma Aldrich (St. Louis, MO, USA). Transcutol ^®^HP CAS. No. 11-90-0; Gattefosse (Saint-Priest, France). Cetyltrimethylammonium bromide (CTAB). CAS. No.57-09-0, Dimethyldidodecylammonium bromide (DDAB). CAS. No.3282-73-3, Soya bean phosphatidylcholine (SPC) CAS.No. 8002-43-5, Carbopol (CP 940); Sigma Aldrich (St. Louis, MO, USA). Hydroxypropyl methylcellulose (methocel) HPMC K4 M. CAS. No. 9004-65-3; Otto Chemie Pvt Ltd., Mumbai, India. Cellulose membrane (12–14 K Da molecular cutoff); SERVA Electrophoresis, Heidelberg, Germany. The bacterial strain *Staphylococcus aureus* ATCC 25923 (*S. aureus*) was obtained through the culture collection of the Regional Centre for Mycology and Biotechnology, Al-Azhar University, Cairo, Egypt. Ampicillin (EPICO Inc., Cairo, Egypt), Tryptone soya broth and agar (TSB; Difco Laboratories, Detroit, MI, USA; Cat. No. 211825, 236950), Mueller-Hinton broth and agar (MHB and MHA; Difco, Detroit, MI, USA; Cat. No. 275730, 225250), ethanol (E. Merck, Darmstadt, Germany; Cat. No. 100983), glycerol (Fluka, Buchs, Switzerland; Cat. No. 56-81-5), glucose (Sigma-Aldrich; Cat. No. G7021), PBS tablets (Sigma-Aldrich, USA; Cat. No. P4417), crystal violet (Winlab, Market Harborough, UK), 96-well flat-bottom polystyrene microplate Greiner Bio-one (Frickenhausen, Germany; Cat. No. 655161) were used.

### 2.2. Formulation of Thymol-Encapsulated Leciplex

Different amounts of soy phosphatidylcholine (SPC) and 10 mg of thymol (THY) were dissolved in 0.5 mL Transcutol HP^®^ in a heated water bath (70 °C); this was the organic phase. The cationic surfactant (either CTAB or DDBA) was dissolved in 9.5 mL of distilled water; this was the aqueous phase. Both phases were mixed at 70 °C under cyclomixing until a uniform yellow dispersion appeared [27].

### 2.3. I-Optimal Design

Prior to the implementation of the I-optimal experimental design, and based on the composition and self-assembly mechanism of Leciplex systems reported in previous studies [21,22,27,28], the lipid-to-surfactant ratio and surfactant type were identified as the most influential formulation factors affecting vesicle formation, particle size, surface charge, and drug encapsulation efficiency. Soy phosphatidylcholine (SPC) was selected as the lipid component because of its excellent biocompatibility and its ability to form stable vesicular bilayers capable of accommodating lipophilic compounds such as THY [29,30]. Cetyltrimethylammonium bromide (CTAB) and Dimethyldidodecylammonium bromide (DDAB) were selected as cationic surfactants owing to their established use in Leciplex systems and their ability to impart a positive surface charge that enhances vesicle stability through electrostatic repulsion [31,32]. It is worth mentioning that DDAB may exhibit concentration-dependent cytotoxicity and irritation when used as a free surfactant in cell culture. DDAB was chosen in the present study because it is one of the most commonly employed cationic surfactants in Leciplex nanovesicles and has been comprehensively reported to enable the formation of stable positively charged nanovesicles. The optimization strategy was based on achieving the smallest possible particle size to facilitate skin permeation [33,34], while simultaneously maximizing entrapment efficiency to ensure adequate THY loading and maximizing zeta potential magnitude to promote colloidal stability [35,36]. The selected formulation variables and their experimental ranges were therefore incorporated into the I-optimal design to systematically investigate their individual and interactive effects on formulation performance and to identify the optimal Leciplex composition. During formulation development, the major challenge was achieving a balance between particle size reduction and maximization of both entrapment efficiency and zeta potential. Formulations with higher lipid content generally showed improved THY encapsulation but larger vesicle size, whereas higher surfactant levels favored particle size reduction. In addition, the investigated surfactants exhibited different performance profiles, where DDAB enhanced entrapment efficiency and surface charge while CTAB tended to produce smaller vesicles. To overcome these formulation trade-offs, an I-optimal design coupled with desirability-based optimization was employed to identify the most suitable combination of formulation variables that simultaneously satisfied all target quality attributes.

I-optimal experimental design was employed to estimate the effects of each design variable on the characteristics of the formulated nanoparticles and to optimize fabrication settings using Design Expert^®^ software 13 (Stat-Ease, Inc., Minneapolis, MN, USA). The variables probed were lipid: SAA ratio (X1) and SAA type (X2), while the responses detected were particle size (PS), zeta potential (ZP), and entrapment efficiency (EE%) as represented in Table 1. Accordingly, optimal conditions for LPX nanovesicles fabrication were established via the desirability value, and the optimization advocated the smallest PS, the largest EE%, and the largest ZP (absolute).

### 2.4. Characterization of Thymol-Leciplex Nanovesicles

#### 2.4.1. Particle Size, Size Distribution, and Zeta Potential

PS, PDI, and ZP of different THY-LPX dispersions were determined using Zetasizer (Nano ZS-90, Malvern Instruments, Southboro, MA, USA) [37]. In brief, 1 mL of each formulation was diluted (10 folds) with distilled water and measured using a scattering angle of 90° at 25 °C.

#### 2.4.2. Entrapment Efficiency (EE%)

The EE% of THY in different THY-LPX dispersions was determined by an indirect method [38,39]. First, THY-LPX dispersions were centrifuged at 15,000 rpm for 1 h at 4 °C in a cooling centrifuge (Hermle Z326K, Wehingen, Germany). Then, the free amount of THY in the clear liquid supernatant was detected using a previously reported and validated UV spectrophotometry method [40]. The % EE of THY was calculated using the following equation:

(1) $$EE\%=\frac{Total\;amount\;of\;THY\;in\;dispersion-Free\;amount\;of\;THY\;insupernatant}{Total\;amount\;of\;THY\;indispersion}\times 100$$

#### 2.4.3. Transmission Electron Microscopy (TEM)

A droplet of the optimized THY-LPX dispersion was diluted, stained with 0.01% (*w*/*v*) phosphotungstic acid, and located on a grid made of carbon and coated with copper and connected to a sample holder. Samples’ fields were photographed using different magnifications [36].

### 2.5. Optimization of the THY-LPX Nanovesicles

The optimized THY-LPX formulation was chosen using Derringer and Suich’s desirability function [41]. Requisite criteria were defined as: minimum PS, maximum EE%, and maximum ZP (absolute value). All responses were conferred equivalent significance (+++). The formulation demonstrating the ultimate overall desirability (D) was recognized as optimal. Desirability was determined as follows [38]:

D = (d1⋅d2⋅…⋅dm)1/m(2)

where D is the total desirability (range: 0–1), dm is the individual desirability of the response, and m is the total number of responses evaluated. The absolute bias percentage between predicted and observed values of the optimized formulation was calculated as [42]:

Bias (%) = [(Predicted − Observed)/Predicted] × 100(3)

### 2.6. Formulation of THY-LPX Gel

The optimized THY-LPX formulation was incorporated into a gel base made of Cp 934 (1.5% *w*/*v*) and HPMC K4m (0.5% *w*/*v*) under mechanical stirring till homogenous viscous gel was obtained.

### 2.7. Characterization of THY-LPX Gel

#### 2.7.1. Physical Appearance and pH

The color and texture of the THY-LPX gel were visually examined. The pH of gel formulation was recorded using a calibrated pH meter (Hanna, model 211, Nusfalau, Romania) at room temperature.

#### 2.7.2. Rheology

Viscosity of the developed THY-LPX gel was determined using a cone and plate type viscometer (Brookfield viscometer; type DVT-2, Brookfield Engineering Labs., Middleboro, MA, USA) at controlled temperatures of 25 °C using a thermostatically controlled water bath, spindle 52 at rotational speed was switched from 10 to 100 rpm to determine gel viscosity.

#### 2.7.3. In Vitro Release

The release of THY from the optimized THY-LPX dispersion and the optimized THY-LPX gel in comparison to the THY aqueous dispersion was studied using the dialysis bag method [43]. Plastic cylindrical tubes were attached to rotating shafts of USP Dissolution Apparatus I to substitute baskets and wrapped by dialysis membrane to close free ends that were in contact with the release medium (50 mL PBS pH 7.4 with 30% ethanol). Test samples were filled in tubes, and the shafts were rotated at 50 rpm at 32 ± 0.5 °C. Samples were withdrawn at specified time intervals and up to 24 h and replaced with an equal volume of fresh medium. Samples were analyzed for THY concentrations using a previously reported, validated UV-spectrophotometric method [40]. The cumulative amount of THY released was calculated and plotted vs time. Kinetics of release were mathematically studied applying zero order, first order, Higuchi model, and Korsmeyer–Peppas model.

#### 2.7.4. Ex Vivo Skin Permeation

The skin permeability of THY from the optimized THY-LPX dispersion and the optimized THY-LPX gel, compared to THY aqueous suspension, was studied in a similar way of release study with minor modification. The dialysis membrane in the release test was replaced with shaved, excised rat skin. Rats were euthanized humanely by CO_2_ inhalation followed by cervical dislocation. Then, the full-thickness dorsal skin was separated, subcutaneous fat was removed, washed with cold saline, and mounted on the free ends of plastic cylindrical tubes [34]. This procedure was approved by the Ethics Committee of the Faculty of Pharmacy, Capital University, Egypt (protocol approval no: IACUC-40A2025, Approval Date: 5 January 2026). Samples inside the tubes were considered the donor compartment, while the surrounding release medium was considered the acceptor compartment. The cumulative amount of THY permeated per unit area (μg/cm^2^) was charted vs time. The steady-state flux (Jss) was determined from the linear portion of the permeation chart by dividing the rate (slope of the linear portion of the curve) by the diffusion area as described in the following equation:

Flux (Jss) = (dM/dt)/A(4)

where Jss is the steady-state flux, (dM/dt)/A is the permeation rate (slope of the linear part of the curve) is (dM/dt; μg/h), and A is the skin diffusion area (cm). The steady-state permeability coefficient (KP; cm/h) was calculated using the following equation:

KP = Jss/Cd(5)

where KP is the permeation coefficient (cm/h), Jss is the steady-state flux, and Cd is the concentration of drugs in the donor compartment (μg/cm^3^).

#### 2.7.5. Differential Scanning Colorimetry (DSC)

Five mg samples of THY, lyophilized optimized gel (optimized TYH-LPX gel), and lyophilized blank LPX gel (LPX-Carbopol/HPMC matrix without thymol) were filled into the aluminum pan of the DSC detector (DSC 50 series, Shimadzu, Kyoto, Japan) after the instrument’s calibration with indium standard. Temperatures of samples were raised gradually at 10 °C/minute between 15 °C and 300 °C in an inert nitrogen atmosphere. Lyophilization was performed using 2% (*w*/*v*) mannitol as a cryoprotectant. The dispersion was pre-frozen using an ultra-low temperature freezer at −80 °C for 24 h, then the samples were lyophilized (Novalyphe NL 500, Halprook, NY, USA), under vacuum at a pressure of 7 × 10^−2^ mBAR, and condenser temperature of −45 °C [44].

#### 2.7.6. Fourier Transform Infrared (FT-IR)

The FT-IR scanning of THY, lyophilized optimized TYH-LPX gel, and lyophilized blank LPX gel was done using an FT-IR spectrophotometer (Vertex80, Bruker Optik GmbH, Ettlingen, Germany). The samples were scanned from 400 to 4000 cm^−1^ at room temperature.

### 2.8. Stability Study

Samples of the optimized THY-LPX nanovesicles and THY-LPX gel were freshly prepared and stored in amber glass vials for 3 months at 4 °C and 25 °C. After the primary analysis of fresh samples (zero time), samples were subjected to repeated analysis after three months of storage. TYH-LPX nanovesicle samples were evaluated for PS, PDI, ZP, and EE%, while THY-LPX gel samples were evaluated in terms of color, phase separation, pH, viscosity, and % release after 24 h to ensure preservation of the gel’s physical stability upon storage. The results were statistically compared to those of the fresh samples.

### 2.9. In Vitro Microbiological Study

#### 2.9.1. Antimicrobial Susceptibility Test and Bacterial Strains

##### Strains and Growth Conditions

*S. aureus* ATCC 25923 was obtained through the culture collection of the Regional Center for Mycology and Biotechnology, Al-Azhar University, Cairo, Egypt. The bacterium was routinely propagated in Tryptone soya broth and agar (TSA; Difco, USA) and incubated at 37 °C for 24 h. For long-term storage, bacterial cultures were kept at −80 °C in glycerol stock solution (20% *v*/*v*). Prior to each experiment, fresh cultures were prepared by subculturing from frozen stocks onto agar plates [45].

##### Agar Diffusion Method

The antimicrobial activity of THY, THY-LPX gel, and blank LPX gel formulations was investigated through an agar well diffusion assay [46]. *S. aureus* was grown overnight on Mueller-Hinton agar (MHA; Difco Laboratories, USA). Three colonies were cultured in 5 mL of Mueller-Hinton broth (MHB) at 37 °C overnight. The bacterial suspension was then adjusted to match a 0.5 McFarland standard (~2 × 10^8^ CFU mL^−1^), then further diluted to 10^6^ CFU mL^−1^. The agar surface was inoculated with 100 μL of the diluted bacterial suspension using a sterile swab. After 15 min of absorption time, a sterilized cork bore was used to produce 6 mm diameter holes in the agar. Each well was then filled with 100 μL of the tested formulations, including THY, THY-LPX gel with (5 mg mL^−1^), blank LPX gel, and standard antibiotics. The positive control was ampicillin (1 µg mL^−1^). The inhibitory zones were measured and reported in millimeters (mm) following a 24 h incubation period at 37 °C. The mean ± standard deviation (SD) was used to express the results of the triple experiment [47].

##### Evaluation of the Minimal Inhibitory and Bactericidal Concentrations for THY and THY-LPX Gel

The broth microdilution test was used to determine the minimum inhibitory concentrations (MICs) of THY, THY-LPX gel, and blank LPX gel, following the Clinical and Laboratory Standards Institute (CLSI) recommendations [48]. *S. aureus* cultures were cultivated overnight at 37 °C on MHA, adjusted to a 0.5 McFarland turbidity, and then diluted in MHB to a final concentration of ~5 × 10^5^ CFU mL^−1^. In sterile 96-well plates with bacterial solution, two-fold serial dilutions of THY and THY-LPX gel were established to achieve concentrations ranging from 512 to 1 µg mL^−1^. Growth control wells contained inoculated broth without antimicrobial agents, while sterility control wells contained broth only. The MIC (µg mL^−1^) has been identified to be the lowest concentration of the test formula that inhibited observable bacterial growth after aerobic incubation at 37 °C for 18–20 h. The minimum bactericidal concentration (MBC) was determined using wells that showed no visible growth in the MIC assay against *S. aureus*. Ten microliters of each clear well were sub-cultured onto TSA plates and incubated for 24 h at 37 °C. Plates were examined for bacterial growth and colony formation following incubation. The lowest concentration that can reduce the bacterial population by at least 99.9% is known as the MBC [49]. The MIC and MBC experiments were performed in triplicate.

#### 2.9.2. The Minimal Biofilm Inhibitory Concentration (MBIC) Test for THY and THY-LPX Gel

The effectiveness of antibiofilm activity for THY, THY-LPX gel, and blank LPX gel against *S. aureus* was assessed using a 96-well flat-bottom microtiter plate assay [50,51]. The bacterial isolate was cultured for 24 h at 37 °C in tryptic soy broth containing 1% (*w*/*v*) filter-sterilized glucose (TSBG). The suspension was diluted (1:100) in fresh TSBG to reach an approximate concentration of 1 × 10^6^ CFU mL^−1^ [52].

A one-hundred-microliter aliquot of the bacterial suspension was then transferred to each well, subsequently adding 100 μL of either THY or THY-LPX gel at concentrations from 128 to 8 μg mL^−1^. The plates were incubated statically at 37 °C for 24 h to allow biofilm formation. Wells containing culture medium only served as the negative control, while wells containing medium with bacterial inoculum but without treatment were considered positive controls.

Following incubation, the supernatant containing planktonic cells was carefully aspirated, and the wells were rinsed twice using phosphate-buffered saline (PBS) to flush out unattached bacterial cells. The plates were then allowed to air-dry before being stained with 1% crystal violet (*w*/*v*) for 20 min. Unbound stain was eliminated by gently washing the plates twice using PBS, followed by air-drying. To solubilize the bound dye, each well received 200 μL of 30% (*v*/*v*) glacial acetic acid. Biofilm biomass was measured at 595 nm using a microplate ELISA reader (BioTek^®^ 800™, Winooski, VT, USA) [53].

The test was carried out in triplicate. The percentage of biofilm inhibition was calculated according to the following equation [11]

Biofilm inhibition (%) is calculated as:

[(optical density of untreated control-optical density of treated)/optical density of untreated control] × 100(6)

The lowest THY concentration that resulted in the greatest degree of biofilm inhibition was identified as the biofilm inhibitory concentration [11].

#### 2.9.3. Biofilm Visualization

##### Scanning Electron Microscopy (SEM)

Biofilm formation was carried out as previously described. Sterilized glass coverslips were introduced in 6-well tissue culture plates containing the bacterial strain suspended in TSBG and treated with either THY or THY-LPX gel at sub-inhibitory concentrations of 32 and 16 μg mL^−1^. Untreated wells served as a positive control. To promote the production of biofilms, the plates were incubated for 24 h at 37 °C. To get rid of any cells that were loosely adhered, the coverslips were carefully rinsed three times with PBS after incubation. After that, the adhering biofilms were fixed for two hours at 4 °C using 2.5% (*v*/*v*) glutaraldehyde prepared in PBS (pH 7.4). A gradual ethanol series (50%, 60%, 70%, 80%, 90%, and 100% *v*/*v*) was used for dehydration, with each stage maintained for 15 min. After air drying, the coverslips were covered with gold using a sputter coater (S150A; Edwards, West Sussex, UK) prior to analysis [54,55]. A field emission scanning electron microscope (FESEM; Quanta FEG 250, Thermo Fisher Scientific, Waltham, MA, USA) was then used to visualize the biofilm structure.

##### Antibiofilm Activity for THY and THY-LPX Gel Using Confocal Laser Scanning Microscopy (CLSM)

In the presence of THY or THY-LPX gel at concentrations of 32 and 16 μg mL^−1^, respectively, biofilms were formed on glass coverslips submerged in TSBG and incubated for 24 h at 37 °C. The coverslips were carefully removed after the incubation period and washed with sterile PBS. The biofilms were stained for 20 min in the dark using a LIVE/DEAD viability kit, which included SYTO9 and propidium iodide (PI) in preparation for CLSM analysis. Before imaging, excess dye was eliminated, and the coverslips were submerged in 10 mM phosphate buffer (pH 7.4). An argon-ion laser at 488 nm was used to excite SYTO9 (green fluorescent), and a He-Ne laser at 543 nm was used to excite propidium iodide (red fluorescent). For SYTO9 and PI, emission signals were collected between 500 and 535 nm and 583 and 688 nm, respectively [56].

### 2.10. In Vivo Study

In vivo therapeutic potential of the optimized TYH-LPX gel against acne was investigated using a rat model for acne [57,58,59]. Animal studies were approved by the Faculty of Pharmacy Ethical Committee, Capital University (protocol approval no: IACUC-40A2025, Approval Date: 5 January 2026).

The in vivo model shows equivalent physiological consequences of acne activation in rats, analogous to those developed in humans [60]. A total of thirty female Sprague Dawley rats (Rattus norvegicus Species) weighing 180–200 g were obtained from the experimental animal unit at the National Cancer Institute, Cairo University. Rats were an outbred strain with no known genetic modification (wild-type genotype). All animals were pathogen-free with a normal immune status (immunocompetent). No previous experimental procedures were performed on the animals before this study. Only animals with normal behavior, intact skin, and no signs of disease were selected. Animals were housed in standard polypropylene cages under controlled environmental conditions (temperature 19–23 °C, humidity 40–65%, and a 12 h light/dark cycle). Rats were fed on standard laboratory chow and water ad libitum. Cages were cleaned regularly to maintain hygiene and prevent infection. To improve animal welfare, environmental enrichment was provided in the form of paper bedding, nesting materials, and tubes. Animals were housed in groups when possible to allow social interaction and reduce stress. The procedure was not painful, so there was no need for analgesics. Animals were monitored daily for signs of pain, severe inflammation, weight loss (>20%), reduced mobility, or distress. The humane endpoint was established to minimize animal suffering. However, none of the animals showed any of the humane endpoints, and no exclusions were made.

#### 2.10.1. Study Design

After one week of acclimatization, rats were allocated to experimental groups using a randomized block design generated with the RAND function in Microsoft Excel. The rats were divided into the following five groups (n = 6/group): GP I, negative control group (normal rats without infection or treatment); GP II, positive control (infected rats without any treatment); GP III, received blank LPX gel (without THY); GP IV received THY-gel; and finally, GP V, treated with optimized THY-LPX gel. Skin inflammation was induced in groups II-V using *S. aureus* (ATCC 2e5923) by intradermal injection of bacteria into the ears of rats. Rats were anesthetized using 4% isoflurane, and then bacterial cultures (50 μL of 1 × 10^7^ CFU) were intradermally injected into the previously shaved skin of the dorsal side of the rats’ left ear. The ear was pre-fixed using double-sided adhesive tape. Likewise, 50 µL of phosphate-buffered saline (PBS 7.4) was injected into the dorsal side of the right ear skin, as a control. One day later, equal volumes of 20 μL of gels (i.e., THY-LPX gel, THY gel (thymol in Carbopol/HPMC matrix), and Blank LPX gel) were applied topically once daily, covering approximately 1 cm^2^ for five successive days to their corresponding rat groups. All experimental procedures and data analyses were conducted in a blinded fashion. The investigator was unaware of group allocation to minimize bias. Also, the investigator responsible for histopathological evaluation and data analysis was blinded to the group allocation of the animals. Treatment groups were coded and then decoded only after completion of the analysis.

#### 2.10.2. Anti-Inflammatory Potential and Ear Thickness

Appearance of inflammation symptoms such as redness, lesions, pustules, or comedones on the skin surface was visually spotted. Moreover, the thickness of rats’ ears was measured before and after treatment using a peacock dial thickness gauge [57].

#### 2.10.3. Histopathological Inspections

Skin specimens were excised from the dorsal region of the rats’ ears and fixed in 10% formalin saline for 24 h. Subsequently, the samples were trimmed, dehydrated through a graded ethanol series, cleared with xylol, and embedded in paraffin wax. Paraffin blocks were sectioned into 6–8 µm thick slices, which were mounted onto glass slides. The sections were then deparaffinized and stained with hematoxylin and eosin (H&E) for histopathological assessment using an electron microscope [61]. Histopathological alterations were semi-quantitatively scored as follows: no abnormality (−), mild changes (+), and moderate changes (++) [62].

### 2.11. Statistical Analysis

All experiments were performed in triplicate, and the obtained data are displayed as mean ± standard deviation. Statistical analysis was performed using GraphPad Prism software (version 10.2.3, Boston, MA, USA). Comparisons between two groups were done using Student’s *t*-test, whereas comparisons among multiple groups were evaluated using one-way ANOVA. Tukey’s post hoc test was implemented to recognize significant differences between group means. A *p*-value ≤ 0.05 was deemed statistically significant.

## 3. Results and Discussion

### 3.1. Model Fit Statistical Analysis

I-optimal design provides a simultaneous examination of experimental factors that affect the characteristics of the synthesized LPX nanovesicles. This research conducted a distinct statistical analysis for all responses to derive a polynomial model that elucidates the relationship between the response and the examined parameters. The optimal polynomial model for each response was selected based on the greatest adjusted R^2^ value and the lowest predicted residual error sum of squares (PRESS) [63].

**Figure 1:**
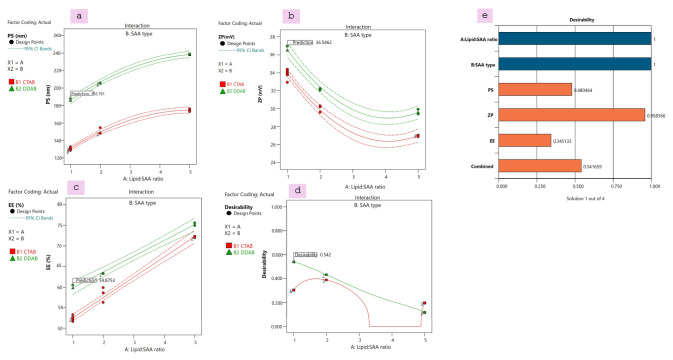
Design Expert^®^ interaction plot for the effect of factors X1: Lipid: SSA ratio and X2: SAA type on design responses, i.e., PS (**a**), ZP (**b**), EE% (**c**), desirability (**d**), desirability and numerical optimization for THY-LPX nanovesicles using I-optimal design; blue bars represent factors and orange bars represent responses (**e**).

Table 1 demonstrates the fit statistical model of the study design, which recommended the quadratic model as the best- fitting model for PS and ZP, while the 2FI model was the best-fitting model for EE%. The adjusted and predicted R^2^ values were in reliable consistency with each other, as the difference between them was less than 0.2 for all the explored responses. In addition, adequate precision values exceeded 4 for all responses, which indicates an acceptable signal: noise ratio. Therefore, the recommended models were the most appropriate for the study of the experimental design space. Table 2 exhibits the thorough formulation variables and the resulting responses. Figure 1 shows the results of the I-optimal design.

#### 3.1.1. Effect of Variables on PS

The vesicle size of different THY-LPX dispersions varied from 128.4 ± 8.1 to 238.87 ± 2.1 nm (Table 2). For this response, the two model variables, X1 and X2, were statistically significant (*p* < 0.0001). Concerning X1 (lipid:SAA ratio), as clearly presented in Figure 1a, increasing lipid:SAA ratio was accompanied by increased particle size; this was a logical outcome as the higher concentrations of the surfactant resulted in enhanced particles’ partitioning and formation of smaller-sized particles and vice versa [33]. Moreover, at higher lipid: SAA ratios, a higher amount of lipids available for nanovesicle assembly led to particle size enlargement. Additionally, the increase in the lipid content elevated the viscosity of the system, promoting the rate of particle aggregation and mass transfer resistances, consequently, a larger particle size [64]. Considering the X2 (SAA type), CTAB-based THY-LPX nanovesicles were substantially smaller in size than those prepared using DDAB (Figure 1a). CTAB is a single-tailed cationic surfactant, while DDAB is a double-tailed cationic lipid [65]. The double-tailed structure of DDAB occupies more volume, leading to the formation of larger vesicles compared to the single-tailed CTAB. Moreover, being smaller in size, with a more flexible headgroup as well as its ability to achieve higher packing density at the interface, CTAB’s single-chain structure allows for tighter packing and better reduction in interfacial tension than the bulky, double-chain structure of DDAB [66]. Consequently, CTAB is more efficient at reducing the interfacial tension between the oil and water phases during the formulation process. Lower interfacial tension led to a greater reduction in surface energy, hence smaller and more stable nanovesicles were obtained. In addition, the reduced affinity between the lipid content of the Leciplex nanocarriers and the more hydrophilic surfactant DDAB (HLB = 18.1) [67] compared to CTAB (HLB = 10) might reduce its solubilization and emulsification effect in the presence of high lipid content, and consequently, larger vesicles were attained [68]. Our results are in line with the previously reported work on similar systems [28,69]. All the synthesized THY-LPX nanovesicles exhibited PDI values ranging from 0.17 to 0.19 (Table 2), indicating homogeneity across all formulations and a narrow unimodal distribution curve of particle size.

#### 3.1.2. Effect of Variables on ZP

The ZP values of different THY-LPX dispersions oscillated between 34.38 ± 0.82 and 26.87 ± 0.81 mV (Table 2), demonstrating that for all formulations, LPX nanocarriers had sufficient charges that would inhibit their aggregation and enhance vesicles’ stability. Cationic surfactants stabilize such nanocarriers predominantly by electrostatic repulsion of similarly charged particles. The more the ZP values, the more repulsion occurs between similarly charged particles, which prevents Ostwald’s ripening and particle aggregation [35]. The observed positive sign of ZP values was attributed to the cationic nature of consumed surfactants CTAB and DDAB. For the ZP model, both X1 and X2 were significant variables (*p* < 0.0001). A high amount of surfactant relative to lipid was associated with dramatic elevation in ZP values as the number of charged molecules of surfactants increased and vice versa (Figure 1b). In addition, DDAB-based nanovesicles were of higher ZP than CTAB-based ones (Figure 1b). This is due to their double-chain cationic lipid structure, which allows for stronger interaction with lipids, better incorporation into the bilayer, and higher lipophilicity than CTAB. This structural difference leads to a more compact, stable cationic surface layer, leading to a higher density of quaternary ammonium head groups per unit area compared to the single-chain surfactant CTAB [28].

#### 3.1.3. Effect of Variables on Entrapment Efficiency (EE%)

Values of EE% of all THY-LPX dispersions ranged between 51.81 ± 0.97 and 75.59 ± 0.97 (Table 2). This moderate entrapment efficiency observed is attributed to the cationic surfactants, which increase membrane fluidity via electrostatic head groups repulsion, resulting in lipid packing defects that enabled the leakage of THY [70]. ANOVA revealed that both X1 and X2 significantly influenced EE% (*p* < 0.0001). Regarding X1, the increased lipid: surfactant ratio had a positive effect on the EE% (Figure 1c). This effect is principally due to the enhanced affinity between the high lipid content and the lipophilic THY. Considering X2, DDAB-based nanovesicles showed higher EE% than CTAB-based ones, obviously due to their double-chain structure, which creates a more hydrophobic, compact, and rigid bilayer environment that better sequesters hydrophobic THY molecules within its core than in the less stable, more fluid structures formed by single-chain surfactants like CTAB. In addition, the quaternary ammonium head group of DDAB provides a strong cationic charge (evidenced by higher ZP values obtained), strengthening the electrostatic stabilization of the vesicles, which in turn leads to a more compact and ordered lipid arrangement. This tight packing reduces the chance of THY leakage.

### 3.2. Selection and Validation of the Optimized TYH-LPX Nanovesicles

The desirability value was used to identify the adequate formulation variables based on the pre-requisite conditions. The selection conditions were minimized PS and maximized ZP (absolute value) and EE%. The optimized THY-LPX made of DDAB and lipid: surfactant ratio 1:1 attained the highest desirability (0.542) with predicted VS (185.791 nm), ZP (36.546), and EE% (59.875), and then, it was selected for further investigations (Table 1) (Figure 1d,e). The absolute bias percentage between predicted and observed values of the optimized formulation did not exceed 10%, confirming the high predictability of the applied statistical model [34].

### 3.3. Evaluation of the Optimized THY-LPX Nanovesicles

#### Transmission Electron Microscopy (TEM)

**Figure 2:**
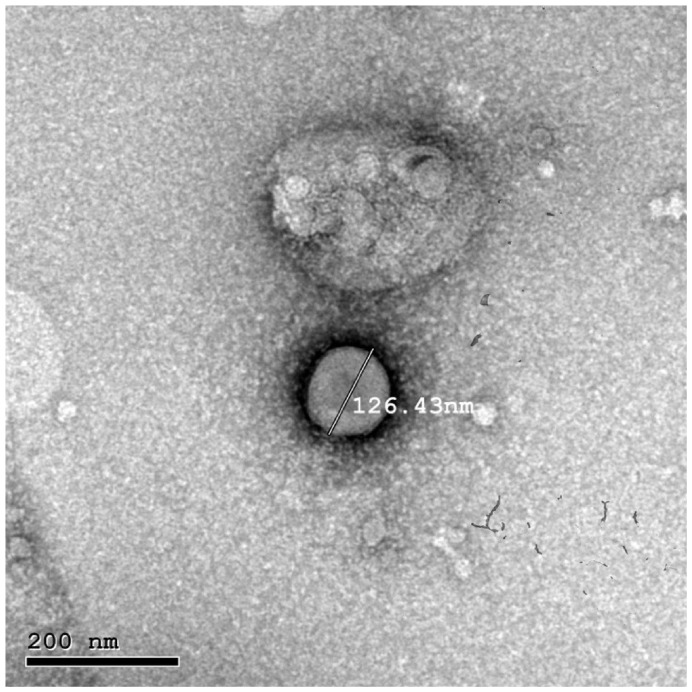
TEM images of optimized THY-LPX nanovesicles.

The TEM photograph of the optimized THY-LPX nanovesicles demonstrated rounded-shaped vesicles with a nanosize (Figure 2). The distinctive bilayer configuration for LPX nanovesicles is recognizable in Figure 2. It is clear that the size detected by TEM was visibly smaller than that detected by DLS. Since a TEM image is obtained for dry samples, DLS detects the hydrodynamic size of dispersed vesicles in liquid form, which are usually larger due to the solvated corona or aggregation. Also, DLS cannot detect the size of individual particles within aggregates. Thus, the sizes obtained by the DLS were larger than those detected by TEM [38].

### 3.4. Characterization of THY-LPX Gel

#### 3.4.1. Physical Appearance and pH

THY-LPX gel was homogeneous, yellow, creamy in texture, and one phase, without any signs of phase separation. This confirmed the uniform distribution of THY-LPX within the gel matrix, which ensures accurate drug content and dose uniformity. The pH value is a necessary aspect of formulations intended for dermal application. The measured value of gel pH was 7.1 ± 0.1. pH values around 5–7 are essentially required for a topical gel to prevent skin irritation [71]

#### 3.4.2. Rheology

**Figure 3:**
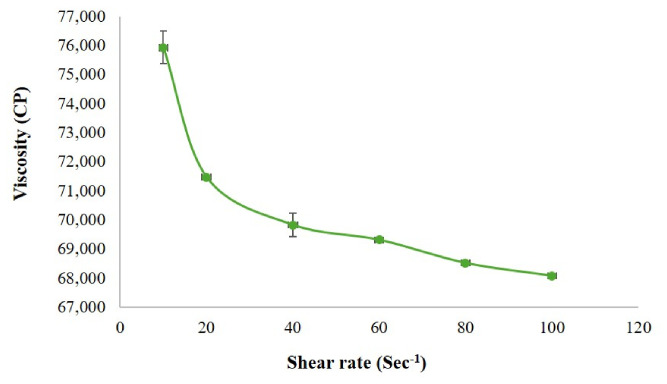
Rheogram of THY-LPX gel represents non-Newtonian shear-thinning flow.

As viewed in Figure 3, THY-LPX gel demonstrated non-Newtonian shear-thinning flow, which is favorable for several reasons. Shear-thinning flow enhances the spreadability of gel and makes its topical application easier [72]. In addition, this type of flow keeps formulation stability and prevents nanovesicle aggregation and further size enlargement upon storage. The shear-thinning (pseudoplastic) rheological behavior of the THY-LPX gel is advantageous for topical administration from a pharmaceutical perspective. Under low shear conditions during storage, the gel’s high viscosity aids in preserving physical stability by reducing nanovesicle sedimentation, aggregation, and phase separation. Conversely, the increased shear forces produced during application and spreading on the skin reduce viscosity, hence promoting uniform dispersion of the formulation throughout the application site and enhancing patient compliance. Post-application, viscosity recovery may extend residence duration on the skin surface, facilitating sustained drug release and improved local drug retention.

#### 3.4.3. In Vitro Release

**Figure 4:**
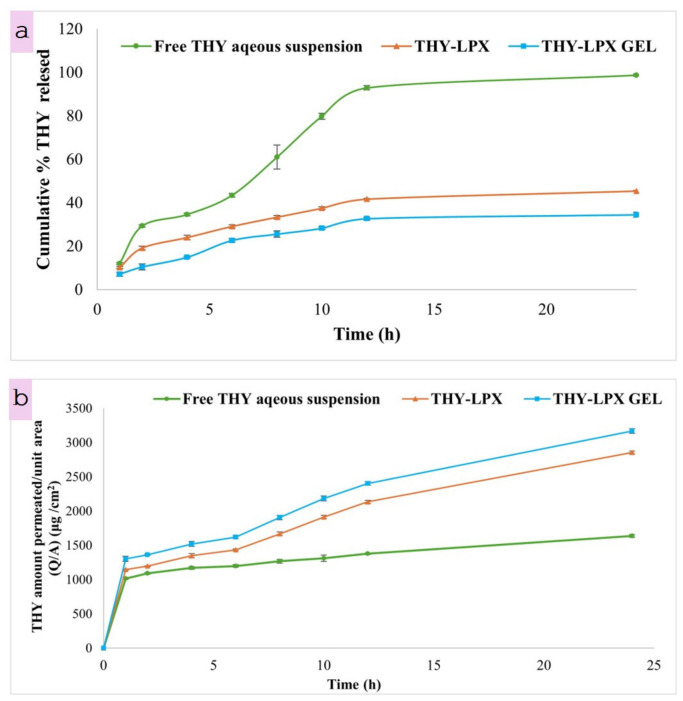
Release profile of THY-LPX nanovesicles and THY-LPX gel vs. THY (**a**). Permeation profile of THY-LPX nanovesicles and THY-LPX gel vs. THY (**b**).

The release profiles revealed formulation-dependent differences among THY, THY-LPX nanovesicles, and THY-LPX gel (Figure 4a). THY showed the fastest release, reaching about 43% within 6 h, with an initial burst of about 30% in 2 h due to rapid dissolution and absence of diffusion barriers. In contrast, THY-LPX exhibited a slower, controlled release (almost 45% over 24 h) with a biphasic pattern, including an initial 19% release at 2 h followed by sustained diffusion through the lipid bilayer, confirming successful encapsulation and stabilization by DDAB. The free drug in the nanosuspension caused an initial burst effect while the drug that was entrapped was released through the nanovesicle, resulting in a slower sustained release during the second phase [73]. The THY-LPX gel demonstrated the slowest release (about 34% over 24 h) due to the additional diffusional resistance of the Carbopol/HPMC polymeric network, with no significant burst effect, indicating efficient vesicle entrapment. Overall, release followed the order: THY > THY-LPX > THY-LPX gel, demonstrating progressive control of drug release. Kinetic analysis showed the best fit to the Korsmeyer–Peppas model, with release exponent (n) values of 0.4714 (THY-LPX dispersion) and 0.5503 (THY-LPX gel), indicating non-Fickian transport governed by both diffusion and polymer relaxation mechanisms [74,75].

#### 3.4.4. Ex Vivo Skin Permeation Study

The ex vivo permeation results showed a trend opposite to the in vitro release, highlighting the importance of formulation design in transdermal delivery (Figure 4b). THY exhibited the lowest permeation (reaching about 1635 µg/cm^2^ at 24 h) with limited steady-state transport (Jss = 33.48 ± 1.46 µg/cm^2^/h; Kp = 0.0335 ± 0.0015 cm/h), due to poor solubility and weak interaction with the stratum corneum. THY-LPX nanovesicles significantly enhanced permeation (2850 µg/cm^2^ at 24 h), with almost 3-fold higher Jss (99.45 ± 0.67 µg/cm^2^/h) and Kp (0.0994 ± 0.0007 cm/h), attributed to lipid-skin interactions, nano-size, and the permeation-enhancing effect of DDAB and Transcutol^®^ [76,77]. This significant improvement is fundamentally based on the nanovesicle-skin interactions and the chemical composition of the nanovesicles. The net negative charge of the stratum corneum at physiological pH generates a significant electrostatic attraction with the positive zeta potential of the cationic surfactant DDAB. The electrostatic attraction extends the formulation’s residence duration and guarantees close contact with the corneocytes. The phospholipid components (lecithin) in the vesicular membrane have significant structural resemblance to the intercellular lipid matrix of the skin. This enables submicron-sized vesicles to intercalate or fuse with the skin’s lipid bilayers, momentarily disturbing their highly organized structure [78]. This fluidization reduces barrier resistance and enhances the entry of THY. Additionally, Transcutol^®^ serves as an effective localized permeation enhancer by expanding the intercellular lipid domains and altering the skin-to-vehicle partition coefficient of the medication [79]. Notably, THY-LPX gel achieved the highest permeation (3150 µg/cm^2^ at 24 h) with superior flux (Jss = 108.74 ± 2.06 µg/cm^2^/h; Kp = 0.1087 ± 0.0021 cm/h), owing to prolonged skin contact, enhanced hydration by the Carbopol/HPMC matrix, and sustained drug reservoir effect. Mechanistically, the Carbopol/HPMC hydrogel network acts as a structural modifier that slows down water evaporation from the skin surface, inducing a localized swelling of the stratum corneum (hyperhydration effect) [80]. This hydration expands the corneocyte gaps, allowing the flexible lipid nanovesicles to penetrate more efficiently. Additionally, the viscosity of the gel matrix alters the thermodynamic activity of the drug, establishing a sustained release ‘depot effect’ within the skin layers that consistently drives the drug downward via a steep concentration gradient [81]. Overall, permeation followed the order: THY-LPX gel > THY-LPX dispersion > THY, confirming a synergistic enhancement by LPX nanovesicles and gel incorporation, and demonstrating that higher release does not necessarily translate to higher permeation.

#### 3.4.5. Differential Scanning Calorimetry (DSC)

**Figure 5:**
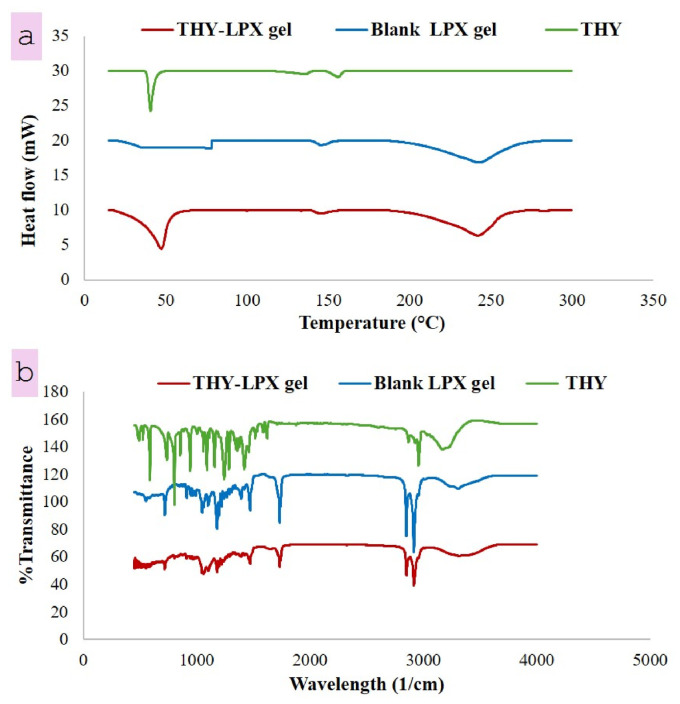
DSC thermogram of THY, THY-LPX gel, and blank LPX gel (**a**). FTIR spectra of THY, THY-LPX gel, and blank LPX gel (**b**).

The DSC thermograms provided valuable insight into the physical state of THY and its compatibility within the developed formulations. In Figure 5a, THY thermogram exhibits a distinct, sharp endothermic peak at approximately 48–50 °C, corresponding to its characteristic melting point, confirming its crystalline nature [82]. The blank LPX gel shows a broad endothermic event centered around 230–250 °C, which can be attributed to polymer dehydration and thermal degradation processes. The absence of any peak in the low-temperature region (40–60 °C) confirms that no interfering components overlap with the melting endotherm of thymol. In the THY-LPX gel formulation, the characteristic melting peak of THY at about 50 °C is markedly diminished and appears significantly broadened with reduced intensity. This attenuation indicates a substantial reduction in thymol crystallinity, suggesting that the drug principally exists in an amorphous or molecularly dispersed state within the formulation. Such behavior is typically associated with successful encapsulation within the lipid nanovesicles and subsequent incorporation into the polymeric gel matrix, which restricts drug recrystallization. Additionally, the preservation of the broad high-temperature endothermic transition (230–250 °C) in the THY-LPX gel, with no significant shift compared to the blank LPX gel, suggests the absence of strong chemical interactions between thymol and the gel-forming polymers. Instead, the interaction is likely physical in nature, involving hydrophobic interactions with lipid components and possible hydrogen bonding within the polymeric network. Overall, the DSC analysis confirms the crystalline nature of THY, its successful transformation into a less crystalline/amorphous state upon formulation, and the compatibility of THY with both the Leciplex nanovesicles and the Carbopol/HPMC gel matrix without evidence of detrimental chemical interactions. This reduction in crystallinity is advantageous, as it can enhance drug solubility, stability, and ultimately its permeation performance and controlled release [83]

#### 3.4.6. Fourier Transform Infrared (FT-IR)

The FTIR spectra provide further evidence regarding the physicochemical state of THY and its compatibility within the developed formulations. The THY spectrum (Figure 5b) shows its characteristic absorption bands [84], including a broad O–H stretching band around ~3200–3500 cm^−1^ [85], C–H stretching vibrations near ~2900 cm^−1^ [86], and distinct aromatic C=C stretching bands in the region of ~1500–1600 cm^−1^, confirming its phenolic structure.

The blank LPX gel spectrum exhibits typical bands of the Carbopol/HPMC matrix, including a broad O–H stretching band (~3200–3500 cm^−1^), C–H stretching (~2900 cm^−1^), and prominent C=O stretching of carboxylic groups around ~1700–1720 cm^−1^ [87], along with C–O–C stretching bands in the ~1000–1300 cm^−1^ region. These peaks represent the polymeric network without interference from THY.

In the THY-LPX gel formulation, the characteristic peaks of thymol are present but appear slightly broadened and reduced in intensity, particularly in the O–H and aromatic regions. No significant peak shifting or disappearance of major functional group bands is observed. This indicates that THY is successfully incorporated within the formulation without undergoing chemical degradation or structural change. Minor broadening and slight attenuation of peaks, especially in the O–H stretching region, suggest possible hydrogen bonding or weak intermolecular interactions between THY, lipid components, and the polymeric gel matrix. However, the absence of new peaks or major shifts confirms that no significant chemical interaction or incompatibility has occurred. Overall, the FTIR analysis supports the DSC findings, confirming successful incorporation of THY in a physically modified (likely amorphous) state, with only weak, non-covalent interactions governing the stability of the THY-LPX gel system.

#### 3.4.7. Stability Study

**Figure 6:**
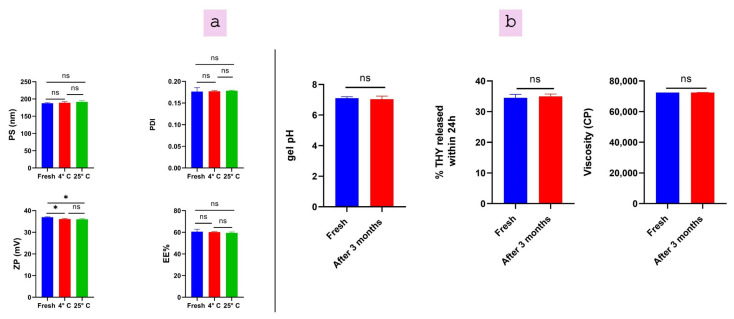
Stability data of optimized THY-LPX nanovesicles (**a**) and THY-LPX gel (**b**), (*); statistically significant difference (*p* < 0.05) and (ns); statistically non-significant difference (*p* > 0.05).

Optimized THY-LPX showed good physical stability, proven by non-significant changes (*p* > 0.05) observed upon retesting of PS, PDI, and EE% after three months os storage at 4 and 25 °C (Figure 6a). Retesting of ZP showed a significant change (*p* < 0.05), and frequent instability was reported for similar systems [38]. The significant decrease in ZP upon the storage of nanovesicles is predominantly driven by the physical degradation of the vesicle membrane, or the adsorption of ions from the medium, which masks the surface charge. During storage, the phospholipids within the vesicles can undergo hydrolysis, resulting in the formation of fatty acids, which sometimes increase the negative charge that, in turn, lowers the magnitude of positive charge conferred by cationic SAA. In addition, the pH of the suspension might change during storage, or ions from the surrounding buffer medium may be adsorbed onto the vesicle surface [88]. This shadows the effective charge of the vesicles, causing the ZP to decrease, which in turn reduces the electrostatic repulsion between vesicles. Moreover, the oxidative degradation of lipids as well as vesicles’ aggregation might also contribute to the decline in ZP. THY-LPX gel also demonstrated excellent physical stability (Figure 6b), evidenced by non-significant change in pH, viscosity, or % THY released within 24 h. The gel also preserved its color and texture upon storage without any phase separation.

### 3.5. Microbiological Studies

#### 3.5.1. Evaluation of Antibacterial Activity

The agar diffusion method was used to assess the antimicrobial activity of THY, THY-LPX gel, and blank LPX gel against *S. aureus*. Both THY and THY-LPX gel were evaluated in comparison to blank gel (Table 3). THY-LPX gel showed the highest activity, represented by zone diameters ± standard deviation.

The bactericidal effect of THY and THY-LPX gel against *S. aureus* is shown in Table 3. After 24 h, the optimal THY-LPX gel demonstrated minimum inhibitory concentration (MIC) and minimum bactericidal concentration (MBC) at a concentration of 156.25 µg·mL^−1^ against *Staphylococcus aureus*, respectively. These values were much lower than the concentration of THY needed to obtain the MIC and MBC values of 312.5 and 625 µg·mL^−1^, respectively. Blank LPX gel showed no activity, while Ampicillin exhibited an MIC value of 1 µg·mL^−1^. These results indicated that nanoencapsulation enhances the antibacterial efficacy of THY. The increased antimicrobial activity associated with encapsulation has also been reported for essential oil components loaded into montmorillonite [89]. For tested strains, including methicillin-resistant Staphylococcus aureus (MRSA) ATCC 43300 and *S. aureus* ATCC 25923, the effective thymol concentration in the nanoemulsion formulation (690 µg·mL^−1^) was lower than that of THY (800 µg·mL^−1^), suggesting improved antimicrobial efficacy achieved through nanoemulsion formulation [90]. The incorporation of THY into nanocarrier systems may mitigate challenges associated with its physicochemical properties, such as volatility, poor water solubility, and instability [26]. Additionally, nanocarriers have been shown to improve topical drug delivery through enhanced protection of encapsulated compounds, prolonged release profiles, greater follicular targeting, and reduced skin irritation [91].

#### 3.5.2. Inhibition of *S. aureus* Biofilm Formation

**Figure 7:**
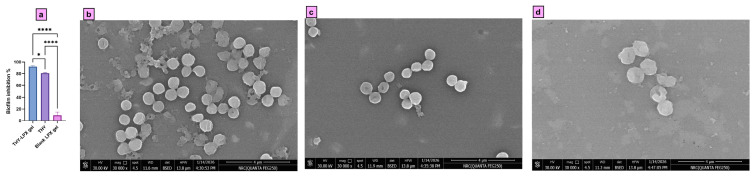
Effect of sub-MIC (¼ MIC) of THY-LPX gel and THY on the biofilm formation of *S. aureus* as evaluated by crystal violet staining (**a**). Data are shown as the percentage of biofilm disruption relative to untreated controls. The Y-axis represents the percentage of biofilm reduction, and error bars represent means ± standard deviation (SD). Statistical difference was determined using one-way ANOVA followed by Tukey’s multiple comparisons test; asterisk refers to statistically significant difference as follows: (*) *p*-value < 0.01, and (****) *p*-value < 0.0001. Scanning electron microscopy image (SEM) of *S. aureus* biofilms treated with THY-LPX gel and THY; control untreated sample showing a dense bacterial biofilm structure on the surface (**b**), THY showing partial biofilm disruption (**c**), and THY-LPX showing biofilm disruption (**d**). Bars = 4 μm.

Biofilm development contributes to antibiotic resistance and the persistence of infections, rendering bacterial communities difficult to eradicate [92]. Crystal violet quantification of biofilm formed in the presence and absence of various concentrations of THY-LPX gel and THY (39–312.5 µg·mL^−1^) demonstrated concentration-dependent antibiofilm activity, with maximum biofilm inhibition of 92.14% at 39 µg·mL^−1^ for THY-LPX gel and 80.80% at 78.12 µg·mL^−1^ for THY without affecting growth. To evaluate the antibiofilm activities of THY-LPX gel and THY, the crystal violet staining assay was used in tissue culture plates. The ability of THY-LPX gel and THY to disrupt preformed, mature biofilms of *S. aureus* without affecting growth was tested at ¼ MIC (Figure 7a). THY-LPX exhibited significant antibiofilm activity against *S. aureus* (*p* < 0.05). THY-LPX gel reduced the biofilm by 92.14 ± 1.52% (n = 3), which was higher than the reduction observed with THY (80.80 ± 1.08%) (n = 3). The blank LPX gel showed negligible inhibition observed (9.22 ± 5.76). Drug delivery in the biofilm depends critically on encapsulation efficiency. Yuan and colleagues found that THY at sub-MIC levels from 64 to 8 µg·mL^−1^ reduced TCH1516 biofilm development in a dose-dependent manner [93]. Concentration inhibits MRSA biofilm formation and disrupts mature MRSA biofilm. THY has also been reported to inhibit up to 88% of MRSA biofilm formation by suppressing the expression of the global regulator sarA, which controls several virulence and biofilm-associated genes [11]. Shin and colleagues showed that at the sublethal concentration (15.6 µg·mL^−1^), the thymol–zinc oxide nanocomposite effectively reduced *S. aureus* biofilm formation by more than 50% [94].

#### 3.5.3. Evaluation of Biofilm Structure Using SEM

The *S. aureus* biofilm’s three-dimensional structure was investigated in the presence and absence of THY and THY-LPX gel at a concentration of ¼ MIC. Untreated biofilms after 24 h showed a dense and well-organized structure, characterized by cell aggregation (Figure 7b). In contrast, treatment with THY (Figure 7c) and THY-LPX (Figure 7d) gel disrupted the biofilm structure. Both of THY and THY-LPX gel reduced cell clustering and decreased the biofilm matrix at concentrations of 78.12 and 39 µg·mL^−1^, respectively. Yuan and colleagues showed that THY treatment at 64 µg·mL^−1^ led to a significant reduction in cell aggregation, with only a limited number of dispersed bacterial cells remaining [94].

#### 3.5.4. Live/Dead Assessment Using CLSM

**Figure 8:**
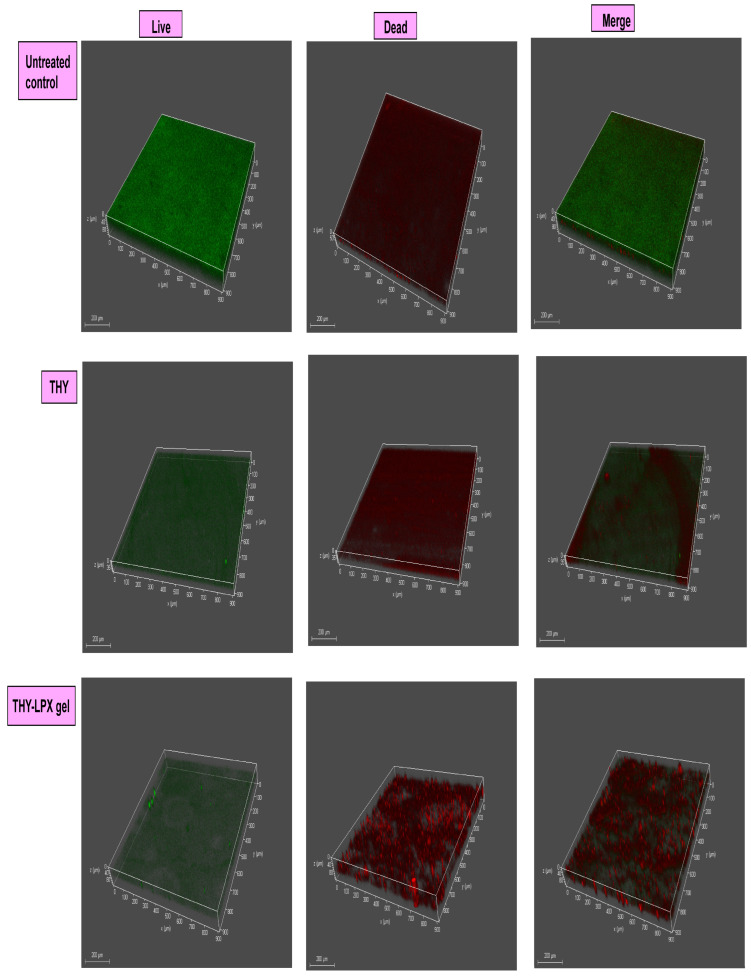
Confocal laser scanning microscopy (CLSM) images of the *S. aureus* biofilms before and after treatment with THY-LPX gel and THY. Fluorescence images of the same samples at 528 nm (green) for SYTO9 signal, 645 nm (red) for PI signal, and merged images are shown. A live and dead cell *S. aureus* was used. The cells were stained with SYTO9 and PI. Bacteria exhibiting red fluorescence are considered dead cells.

Bacteria in biofilms have growth patterns that enhance their ability to evade host immune defenses. Biofilm structure has limited nutrient availability, oxygen depletion, and accumulation of metabolic byproducts, which can drive bacterial cells to starve and enter a non-vegetative state, reducing their susceptibility to antimicrobial agents [95]. The killing effect of THY and THY-LPX gel on *S. aureus* biofilm was evaluated using fluorescence analysis. In this experiment, SYTO9 and PI interact with nucleic acids and differentiate live and dead cells, which are visualized as green and red fluorescence, respectively. As shown in Figure 8, treated and untreated biofilms differ clearly. Biofilms treated with THY showed predominantly green fluorescence with a limited red signal, suggesting that most bacterial cells remained viable. THY-LPX gel-treated biofilms showed a strong red fluorescence, indicating a significant reduction in viable cells. Untreated biofilms displayed intense green fluorescence, confirming the predominance of live bacterial populations.

### 3.6. In Vivo Study

Acne is a common chronic inflammatory skin condition affecting a large number of the population, particularly adolescents and young adults. The pathogenesis of acne is multifactorial, associated with factors including increased sebum production, follicular hyperkeratinization, bacterial colonization, and release of inflammatory mediators [1]. Animal models were used to better understand the pathophysiology of acne and to evaluate potential therapeutic agents [96]. Sprague Dawley (SD) rats are among the most widely used outbred laboratory rat models in biomedical research. Their prominence is largely due to their well-characterized biology, relative ease of handling, and specific physiological and immunological similarities to humans, particularly in studies involving chronic disease and inflammatory responses [97,98]. The use of Sprague Dawley mimics acne-like skin inflammation and allows us to understand the acne pathogenesis and its potential translation to the human condition, offering a valuable platform to study the four key pathogenic factors of human acne: hyperseborrhea, follicular hyperkeratinization, bacterial colonization (*S. aureus* or *C. acnes)*, and inflammation [99,100].

#### 3.6.1. Anti-Inflammatory Potential and Ear Thickness

The inflammatory signs could be recognized as redness and an obvious increase in skin thickness of the infected ear in all groups. *S. aureus* skin infection usually results in skin colonization and contamination that may trigger further infections affecting both superficial and deeper layers of the skin. This invasive process typically begins when the skin barrier is disrupted, leading to the formation of microthrombi. *S. aureus* expresses adhesins, surface proteins with strong affinity for fibrin, fibrinogen, and collagen, facilitating its attachment [101]. Additionally, *S. aureus* produces coagulases that enhance thrombus formation. Bacterial proteases contribute to clot degradation, enabling further tissue penetration [102]. These enzymes also impair complement-mediated opsonization and reduce phagocytic activity. Moreover, *S. aureus* releases proteins that interact with inflamed endothelial cells, promoting bacterial adhesion and ultimately leading to endovascular infection [102].

Acne vulgaris also involves chronic inflammation with increased inflammatory cytokines, including IL-1β, TNF-α, IL-6, and IL-8 [103,104]. Interestingly, previous studies demonstrated the anti-inflammatory and antibacterial activities of thymol, particularly via inhibition of proinflammatory cytokines, IL-1β, TNF-α, and IL-6 in a mouse model of acute lung injury [105] and microglial inflammation [106]. Mechanistically, the anti-inflammatory effect of thymol potentially involves modulation of MAPK, NF-*κ*B, JAK/STAT, and arachidonic acid signaling pathways [105].

**Figure 9:**
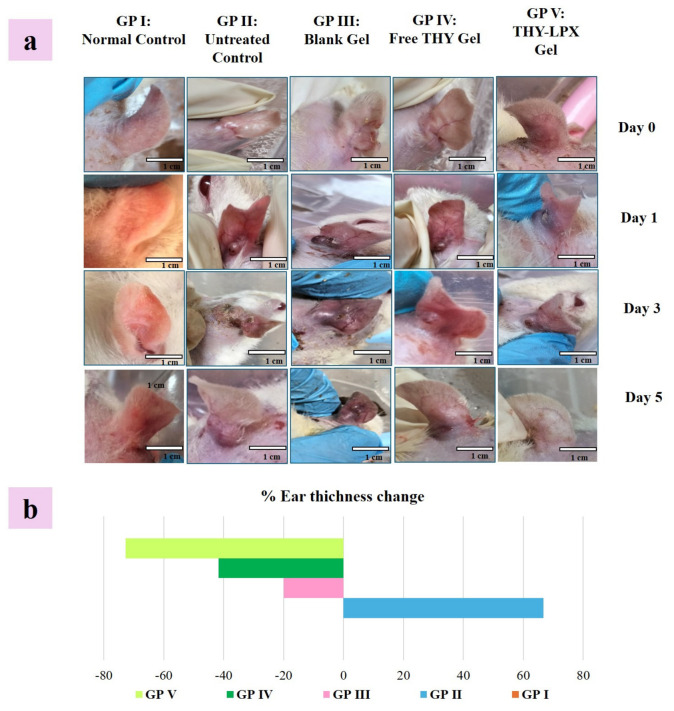
Macroscopic images of rat ears over 5 days after infection with *S. aureus*, followed by treatment with THY, optimized THY-LPX gel, or blank LPX gel, compared to normal control and untreated control. All images were captured at a fixed distance and include a 1 cm scale bar for size reference (**a**). Bar chart of %ear thickness change among all groups (**b**).

From Figure 9a, groups IV and V demonstrated a substantial decrease in inflammatory signs on the fifth day of treatment compared to normal (GPI), negative control (GP II), and Blank (GP III). However, group V (THY-LPX gel) exhibited complete recovery by day 5 without any visible inflammatory signs or pustules, compared to group IV, which demonstrated slight skin redness by day 5. Additionally, ear thickness was reduced by 72.7% following THY-LPX gel treatment (GP V) compared to 41.7% following THY gel treatment (GP IV), 20% following blank gel treatment (GP III), and 0% for normal control (GP I) by day 5 (Figure 9b). The enhanced therapeutic efficacy of THY-LPX gel relative to free THY gel indicates that the LPX formulation improves THY transport to the S. aureus infection site. This enhancement is mostly ascribed to improved skin penetration and prolonged local release of THT, rather than a change in THY’s inherent antibacterial or anti-inflammatory properties. Leciplex enhances the partitioning of THY into the stratum corneum and extends medication retention within skin layers, presumably contributing to the expedited and more thorough resolution of inflammation [107].

On the other hand, the untreated control group (GP II) showed an extensive increase in ear thickness (66.7%) by day 5 that was attributed to the worsened inflammatory response in the absence of any treatment. Former reports suggest the incidence of dermonecrosis caused by *S. aureus* during skin and soft tissue infections, which are almost exclusively caused by secreted bacterial toxins. This dermonecrosis results in excessive inflammation, which further leads to skin damage [21]. Moreover, these secreted proteins have cytolytic influence on leukocytes that act as an immune evasion mechanism for *S. aureus*, thus facilitating persistence of the infection and increased skin thickness as observed in group II.

#### 3.6.2. Histopathological Examination

**Figure 10:**
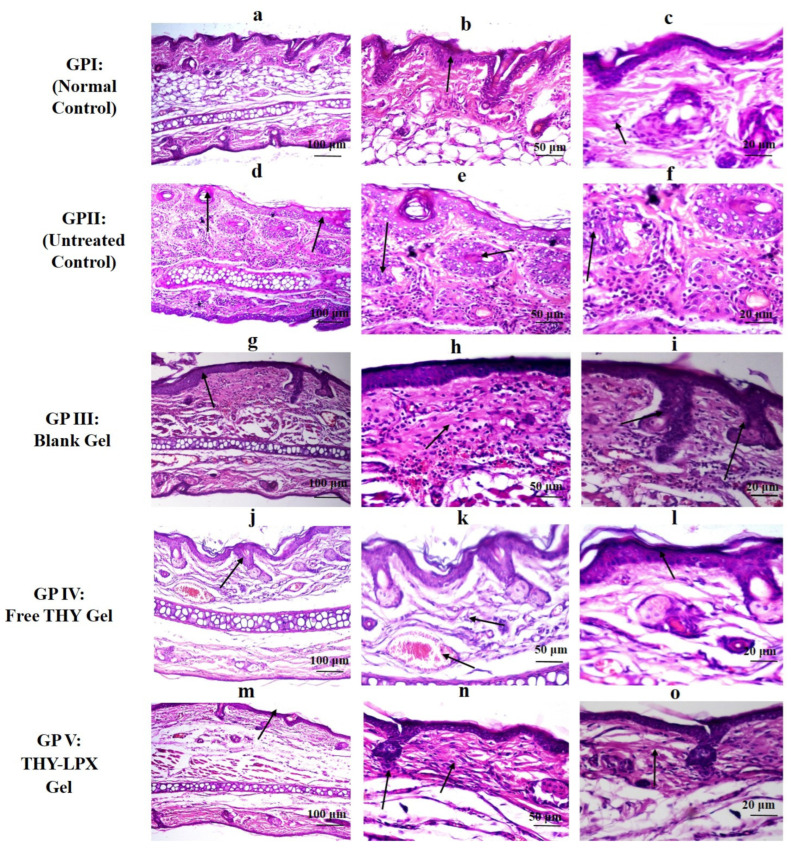
GP I Photomicrograph of ear tissue section showing: (**a**)—normal histological structure of rat ear skin, (**b**)—thick epidermal layer covered with deeply eosinophilic keratin arrow, (**c**)—dermal layer composed of dense fibrous tissue, cartilaginous layer, and a few blood capillaries arrow. GP II Photomicrograph of ear tissue section showing: (**d**)—hyperacanthosis and hyperkeratosis arrow, (**e**)—dense horn that expands the infundibulum and extends into the sebaceous ducts arrow, (**f**)—abscesses, edema, and congestion of dermal blood capillaries arrow. GP III Photomicrograph of ear tissue section showing: (**g**)—hyperacanthosis and hyperkeratosis arrow, (**h**)—leukocytic infiltration mainly neutrophils and lymphocytes arrow, (**i**)—A cumulation of dense horn that expands the infundibulum and extends into the sebaceous ducts arrow. GP IV Photomicrograph of ear tissue section showing: (**j**)—dilation of the hair follicle infundibulum arrow, (**k**)—mild edema and dilatation of the blood vessels arrow, (**l**)—Mild hyperacanthosis and hyperkeratosis arrow. GP V Photomicrograph of ear tissue section showing: (**m**)—Mild epithelial hyperplasia arrow, (**n**)—Small number of inflammatory cells infiltration arrow, (**o**)—Mild edema of dermal stroma arrow.

Animals were typically euthanized using an overdose of isoflurane. The skin tissue segment of the control group (normal animals, GP I) exhibited a typical histologic structure of rat ear skin. The ear’s skin is distinguished by a thick epidermal layer, which is covered by a deep eosinophilic keratin layer, and lacks a horn in the infundibulum. The dermal layer consists of thick fibrous connective tissue, a cartilaginous layer, and a limited number of blood capillaries, devoid of any inflammatory response (Figure 10 GPI (a-b-c)). In contrast, the ear skin of rats infected with *S. aureus* (infected group; GP II) exhibited hyperacanthosis, hyperkeratosis, and the formation of thick keratin, which expanded the infundibulum and extended into the sebaceous ducts. Significant inflammatory responses were seen, accompanied by extensive cellular infiltration, mostly polymorphonuclear leukocytes (PMNL) and macrophages at the interface of the dermis and subcutis. Additionally, abscesses around the infundibulum that may lead to the formation of papules or pustules were seen. Edema and congestion of cutaneous blood capillaries were also seen (Figure 10 GPII (d-e-f)). The group that received the blank gel (GPIII) exhibited a minor improvement relative to the untreated group (GPII). Smears from this group exhibited hyperacanthosis and hyperkeratosis, characterized by the buildup of thick keratin (horn) that enlarges the infundibulum and spreads into the sebaceous ducts. The dermal layer exhibited leukocytic infiltration, mostly consisting of neutrophils and lymphocytes, along with edema and significant dilation of the blood capillaries. The dermis had a remarkable erythrocyte extravasation and abscesses (Figure 10 GP III (g-h-i)).

The THY gel-treated group (GPIV) exhibited moderate improvement compared to normal, control, and blank gel-treated groups. Mild hyperacanthosis and hyperkeratosis with a significant decrease in inflammatory cell infiltration and dilation of the hair follicle infundibulum were observed. Additionally, the dermal layer revealed mild edema and dilatation of the blood vessels (Figure 10 GP IV(j-k-l)). Finally, the THY-LPX gel-treated group (GPV) revealed significant improvement. Smears showed mild epithelial hyperplasia and a dense, consistent accumulation of horns that is stretching the follicular canal. The presence of a small number of inflammatory cells, especially around the follicles or sebaceous ducts, with mild edema of the dermal stroma was also obvious (Figure 10 GP V (m-n-o)).

Collectively, THY-LPX gel demonstrated promising results as a topical acne therapeutic system owing to several attributes; i.e., nanosizing strategy that enhanced dermal permeation, LPX system composed of SPC that has excellent biocompatibility, and its ability to form stable vesicular bilayers capable of accommodating lipophilic compounds such as THY [29,30] and DDAB that was selected as a cationic surfactant because of its established use in Leciplex nanocarriers and its ability to generate highly positively charged, physically stable nanovesicles. Owing to its double alkyl-chain structure, DDAB exhibits strong bilayer incorporation and provides enhanced encapsulation of lipophilic molecules compared with single-chain cationic surfactants. Although DDAB has been reported to exhibit concentration-dependent cytotoxicity when used in its free form [108,109], its incorporation within phospholipid-based nanovesicles and further incorporation into Carbopol/HPMC matrix limit direct cellular exposure while preserving its beneficial stabilizing and permeation-enhancing properties. Moreover, no signs of local irritation or histopathological abnormalities attributable to the formulation were observed during the in vivo evaluation conducted in the present study, further confirming the safety and biocompatibility of the developed formulation.

## 4. Conclusions

Cationic Leciplex nanovesicles successfully enhanced the topical delivery and therapeutic performance of THY. The optimized THY-LPX system (SPC: DDAB; 1:1) exhibited favorable nanoscale characteristics, potent antibacterial activity, and effective biofilm disruption. Incorporation into a Carbopol/HPMC gel further improved skin retention and permeation, translating into a pronounced in vivo anti-inflammatory effect and histological recovery. Collectively, the developed THY-LPX gel represents a promising, multifunctional topical platform for acne management, combining antimicrobial, antibiofilm, and anti-inflammatory actions. Although the current in vivo model provides useful evidence of the efficacy of the THY-LPX system, it does not fully recapitulate the complexity of acne vulgaris pathology, including host-microbe interaction, recurrent inflammation, and cytotoxicity studies. Thus, further investigations are warranted to support the clinical application. Although the formulation demonstrated promising antimicrobial activity against *Staphylococcus aureus*, the activity against *Cutibacterium acnes*, a major pathogen implicated in acne vulgaris, was not evaluated in the present study. Future investigations should assess the efficacy of the formulation against *C. acnes* under appropriate anaerobic culture conditions to further establish its potential for acne management. Moreover, long-term dermal safety, skin irritation, and sensitization studies following repeated topical application will be evaluated in future in vivo investigations. In addition, comprehensive stability studies following ICH Q1A(R2) guidelines remain necessary to establish the long-term physicochemical and microbiological stability of the formulation.

## Figures and Tables

**Table 1:** I-optimal design highlighting different levels of independent variables, targeted optimization criteria for the responses, and the summarized design’s statistical analysis.

**Independent Variables**	**Constraints**		
X_1_: Lipid:SAA ratio	1:1	2:1	5:1
X_2_: SAA type	CTAB	DDAB	
**Responses**	**Optimization Target**		
Y_1_: PS	Minimize		
Y_2_: ZP	Maximize (absolute values)		
Y_3_: % EE	Maximize		
**Statistical Summary**	**Y1: VS (nm)**	**Y2: ZP (mV)**	**Y3: EE%**
Minimum	128.4	26.8667	51.5956
Maximum	238.867	36.9667	75.5855
Model	Quadratic	Quadratic	2FI
Model *p*-value	<0.0001	<0.0001	<0.0001
Model F-Value	946.25	123.46	328.74
Adequate precision	78.6064	34.5018	42.1525
R^2^	0.9974	0.9802	0.9890
Adjusted R^2^	0.9963	0.9722	0.9860
Predicted R^2^	0.9937	0.9545	0.9840
Significant variables	X_1_, X_2_	X_1_, X_2_	X_1_, X_2_
Predicted values	185.79	36.55	59.88
Observed value	187.70	36.97	60.51
% Bias	1.02	1.14	1.04

**Table 2:** Formulation variables of THY-LPX nanovesicles and their obtained responses.

Runs	Variables		Responses (Mean ± SD)			PDI
	X1: Lipid:SAA	X2: SAA Type	PS (nm)	ZP (mV)	EE%	
1	2:1	CTAB	154.533 ± 3.98	+30.3033 ± 0.78	59.8753 ± 1.70	0.189 ± 0.177
2	1:1	CTAB	131.54 ± 7.23	+34.3767 ± 0.82	52.2325 ± 0.64	0.172 ± 0.18
9	1:1	DDAB	187.7 ± 1.87	+36.9667 ± 0.21	60.5122 ± 2.29	0.186 ± 0.18
10	5:1	CTAB	172.7 ± 2.34	+27.0333 ± 0.42	71.9764 ± 1.10	0.183 ± 0.180
17	2:1	CTAB	148.3 ± 3.84	+30.2033 ± 0.4	56.2662 ± 0.37	0.184 ± 0.17
8	2:1	DDAB	205.5 ± 2.35	+32.0667 ± 0.57	63.2721 ± 0.37	0.172 ± 0.18
11	1:1	CTAB	130.673 ± 9.67	+34.0767 ± 1.7	51.8079 ± 0.97	0.185 ± 0.18
4	2:1	CTAB	148.2 ± 6	+29.6 ± 0.75	58.6015 ± 1.10	0.175 ± 0.17
12	2:1	DDAB	205.3 ± 4.75	+32.2333 ± 0.38	63.2721 ± 0.74	0.182 ± 0.18
14	5:1	CTAB	176.1 ± 4.53	+26.8667 ± 0.814	71.9764 ± 0.64	0.183 ± 0.18
6	5:1	DDAB	238.133 ± 2.5	+29.9333 ± 1.33	74.9486 ± 0.97	0.175 ± 0.18
13	1:1	CTAB	128.5 ± 6.1	+34.25 ± 1.78	52.2325 ± 0.64	0.175 ± 0.18
7	5:1	DDAB	238.867 ± 2.1	+29.4 ± 0.61	75.5855 ± 0.97	0.179 ± 0.97
16	1:1	CTAB	128.4 ± 8.11	+33.7567 ± 1.51	53.294 ± 0.97	0.177 ± 0.18
15	1:1	CTAB	132.533 ± 11.6	+32.9333 ± 1.42	51.5956 ± 1.27	0.174 ± 0.172
3	2:1	CTAB	154.533 ± 3.98	+30.3033 ± 0.78	59.8753 ± 1.7	0.189 ± 1.7
5	1:1	CTAB	131.54 ± 7.23	+34.3767 ± 0.82	52.2325 ± 0.64	0.172 ± 0.18

**Table 3:** Diameters of inhibition zones (mm), MIC, and MBC for THY, THY-LPX gel, and blank LPX gel against *S. aureus.*

	Inhibition Zone (mm)	MIC µg·mL^−1^	MBC µg·mL^−1^
THY	32 ±1	312.5	625
THY-LPX gel	40.7 ±1.15	156.25	156.25
Ampicillin (positive control)	26.3 ± 1.53	1	1
Blank LPX gel	No effect	No effect	No effect

## Data Availability

The original contributions presented in this study are included in the article/Supplementary Materials. Further inquiries can be directed to the corresponding authors.

## Supplementary Materials

The following supporting information can be downloaded at: https://www.mdpi.com/article/10.3390/pharmaceutics18070795/s1.
