# Supplementary material for: Leciplex Nanocarriers: An Optimized Platform for Thymol Delivery in Acne Management
Source: Pharmaceutics. 2026 Jun 28;18(7):795. doi: 10.3390/pharmaceutics18070795 (PMC13414981; doi:10.3390/pharmaceutics18070795)
Supplement: Supplementary file 1 [file pharmaceutics-18-00795-s001.zip › pharmaceutics-4364639-supplementary.pdf]

Supplementary Materials

Leciplex Nanocarriers: An Optimized Platform for Thymol Delivery in Acne Management

Soha Elsalhy, Norhan Tantawy, Eman E. El Naggar, Wesam E. Gawad,Amira M. Badr, Reem T. Atawia, Jihad Mahmoud Alsofany

A. Confocal Laser Scanning Microscopy (CLSM) Raw Figures

1-Untreated live

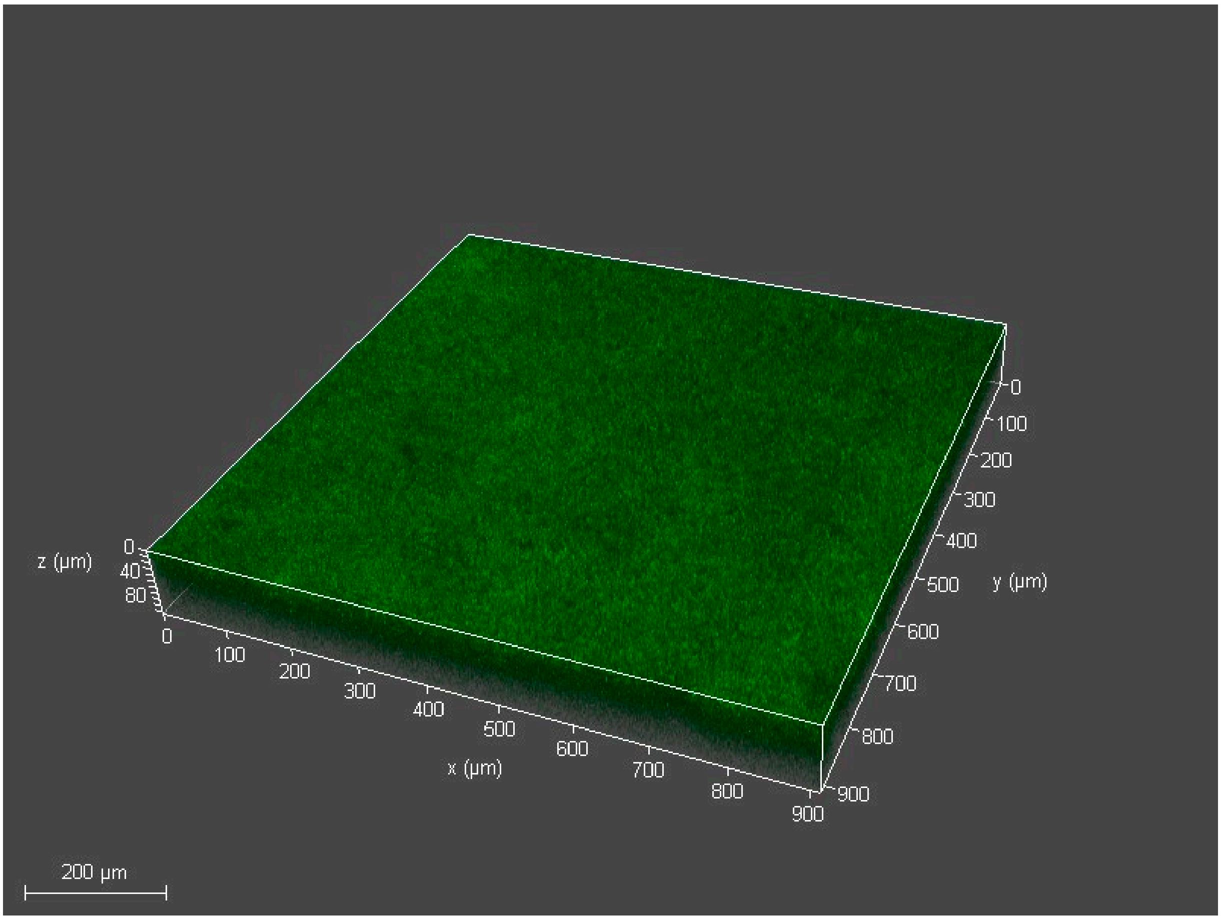

2-Untreated merge

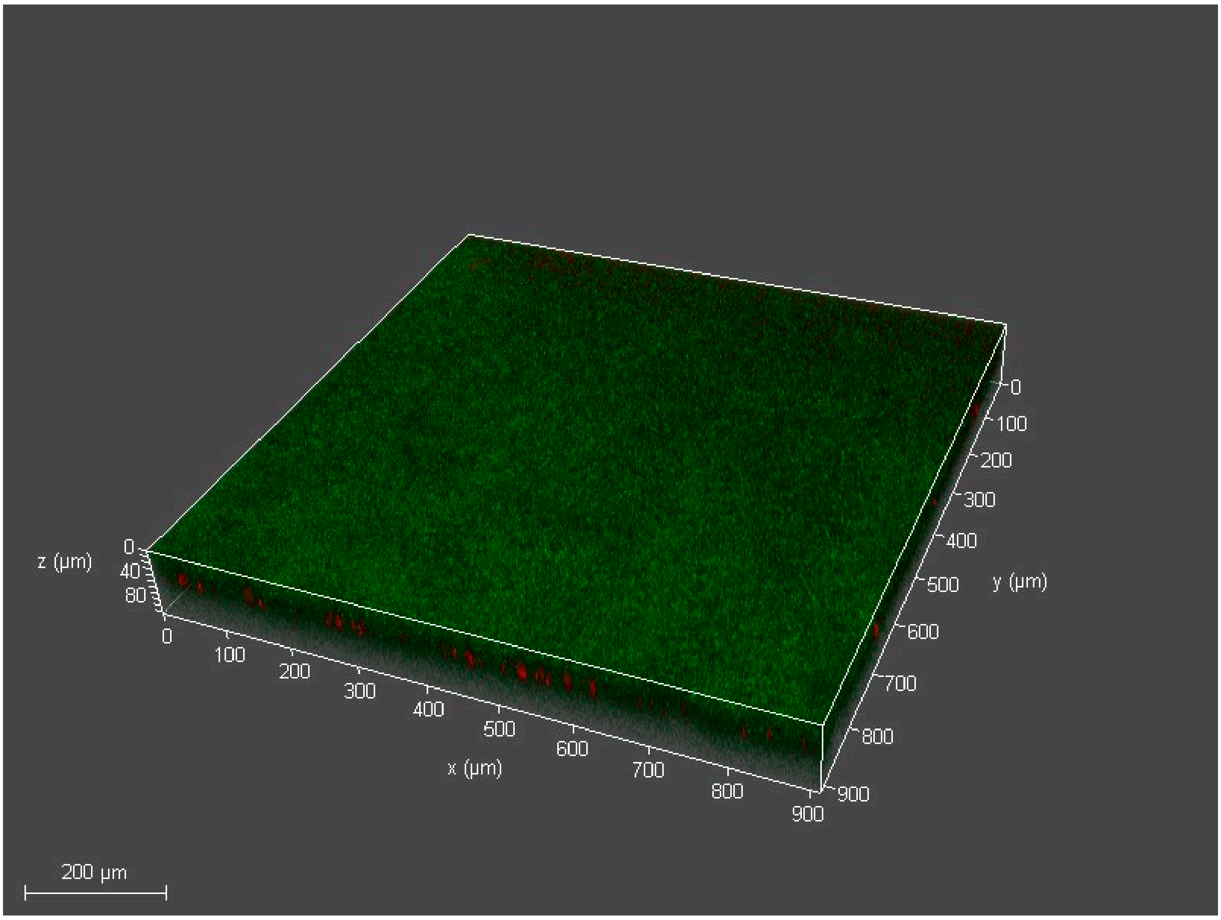

3-Untreated dead

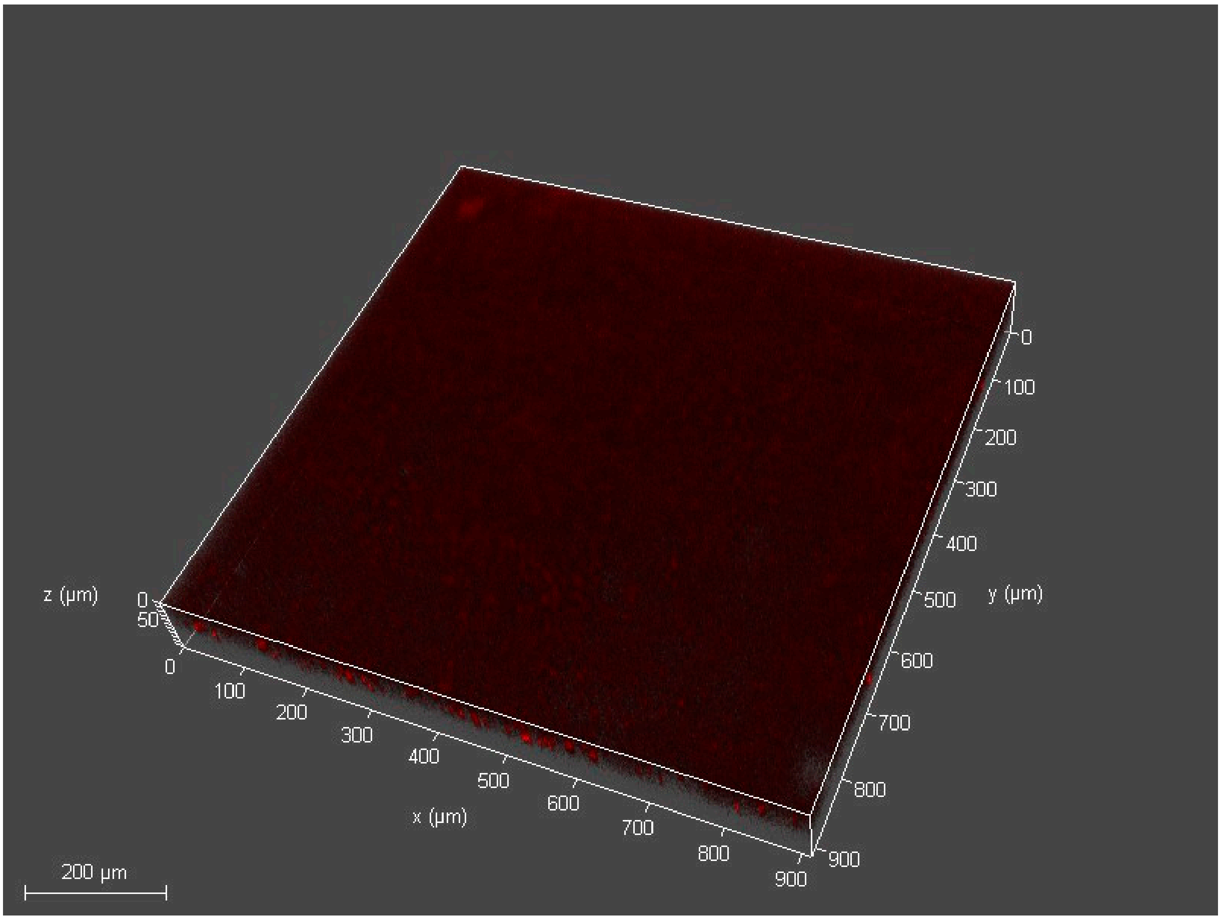

4-THY LPX live

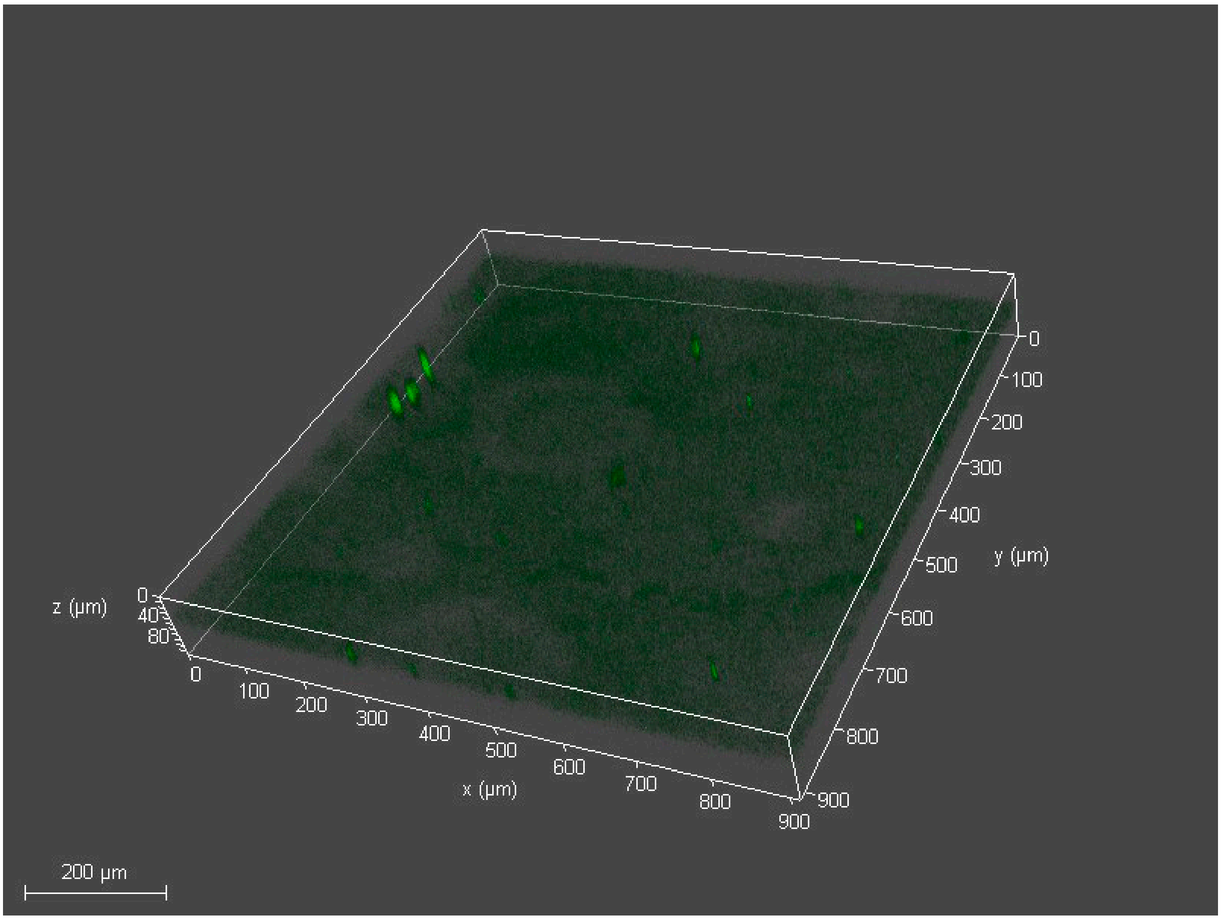

5-THY-LPX dead

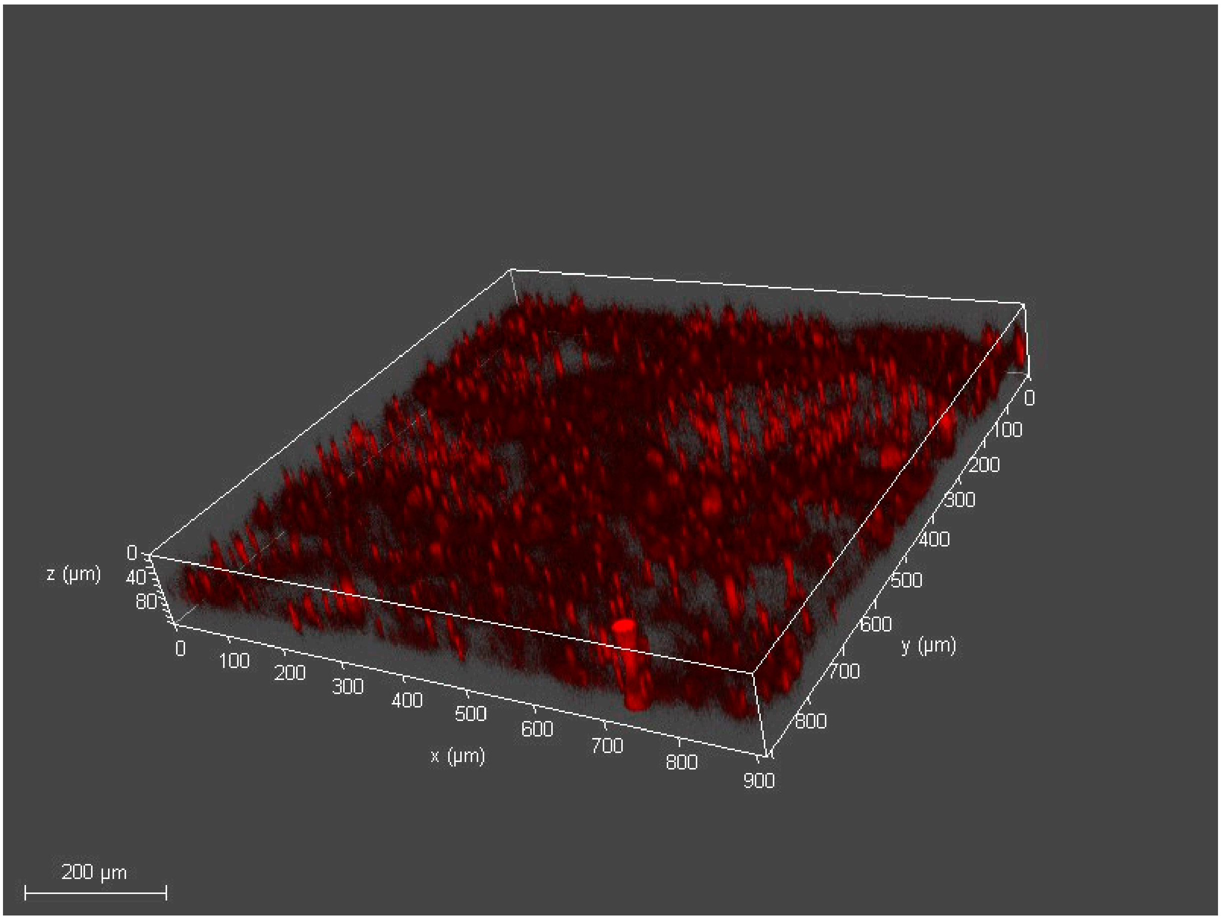

6- THY-LPX merge

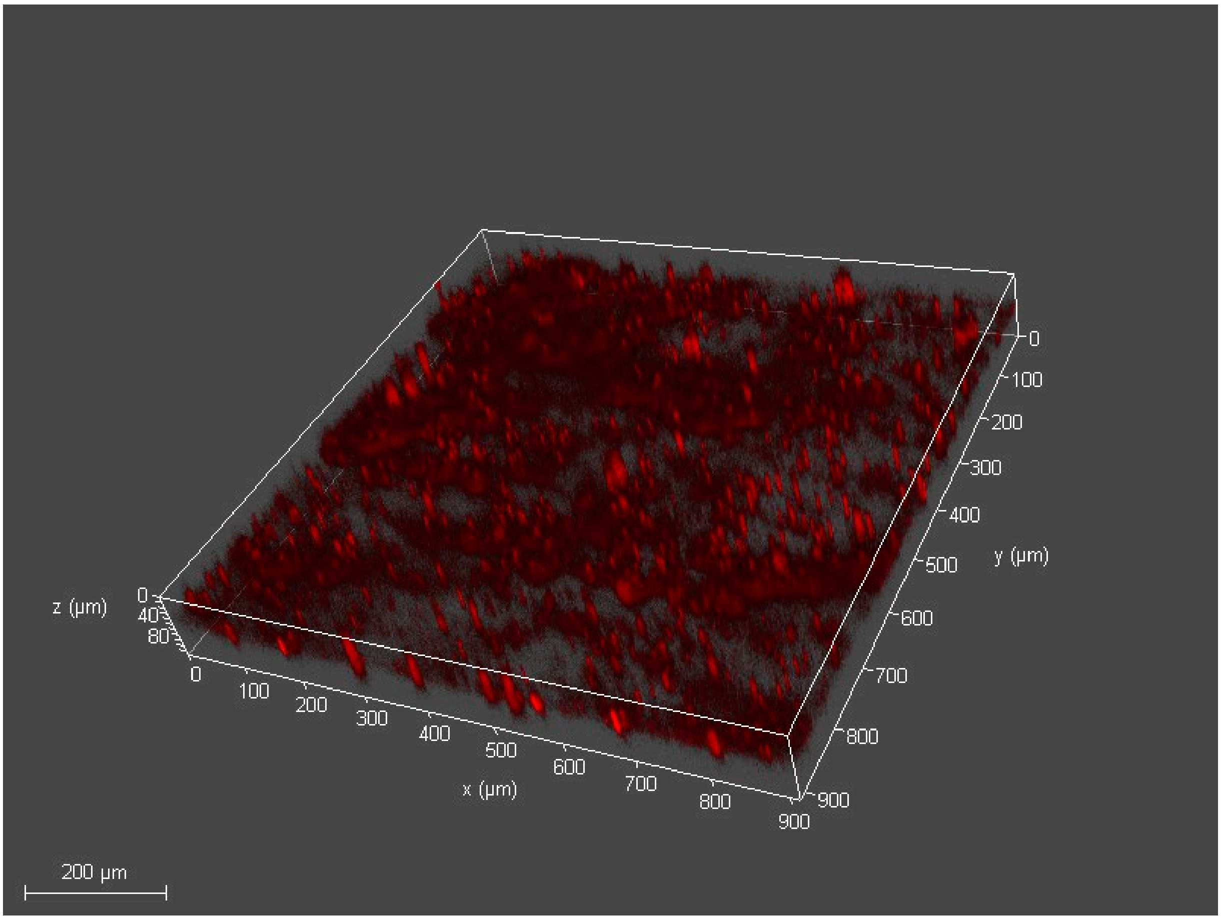
z (µm)
0
40
80
0
100
200
300
400
500
600
700
800
900
x (µm)
0
100
200
300
400
500
600
700
800
900
y (µm)
200 µm

7- THY dead

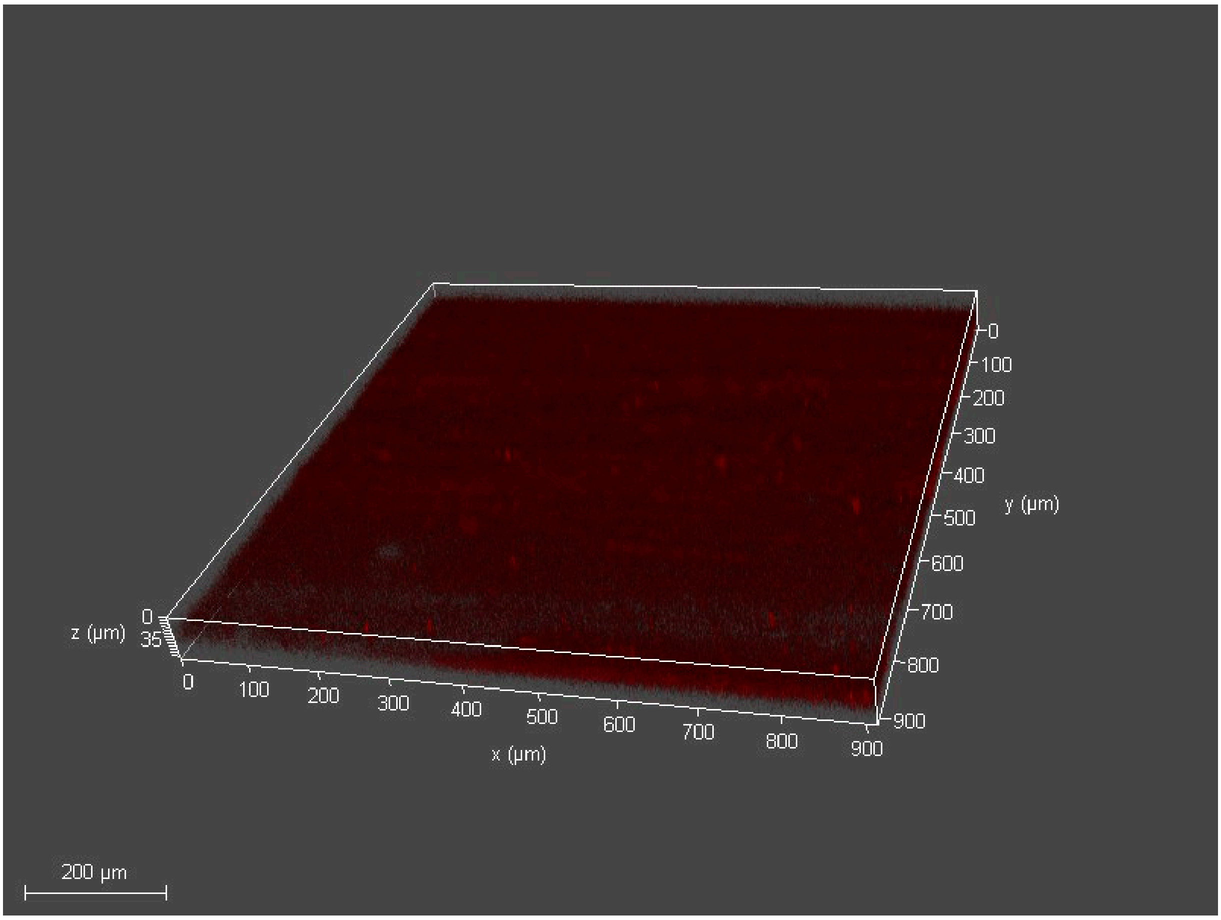

8- THY live

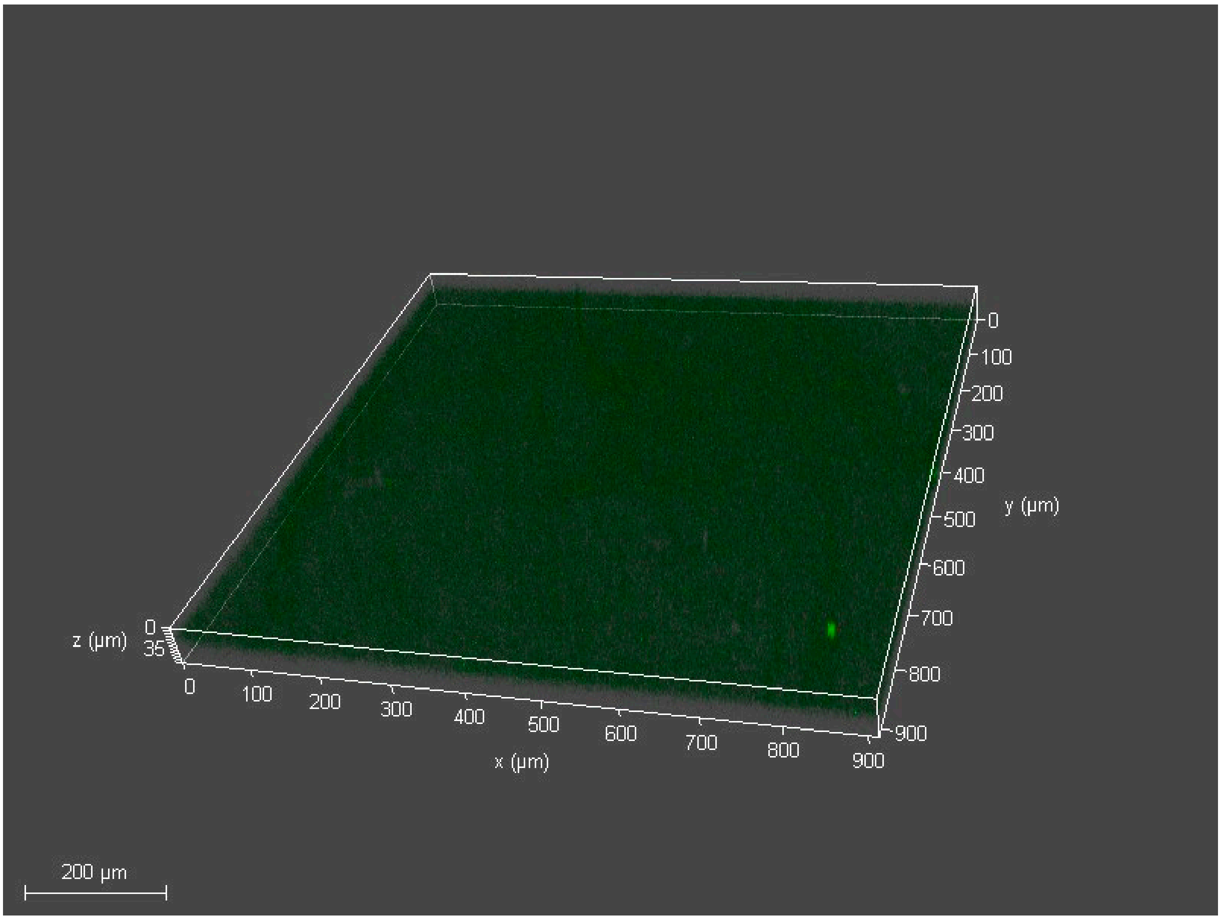
0
100
200
300
400
500
600
700
800
900
y (µm)
z (µm)
0
35
0
100
200
300
400
500
600
700
800
900
x (µm)
200 µm

9- THY merge

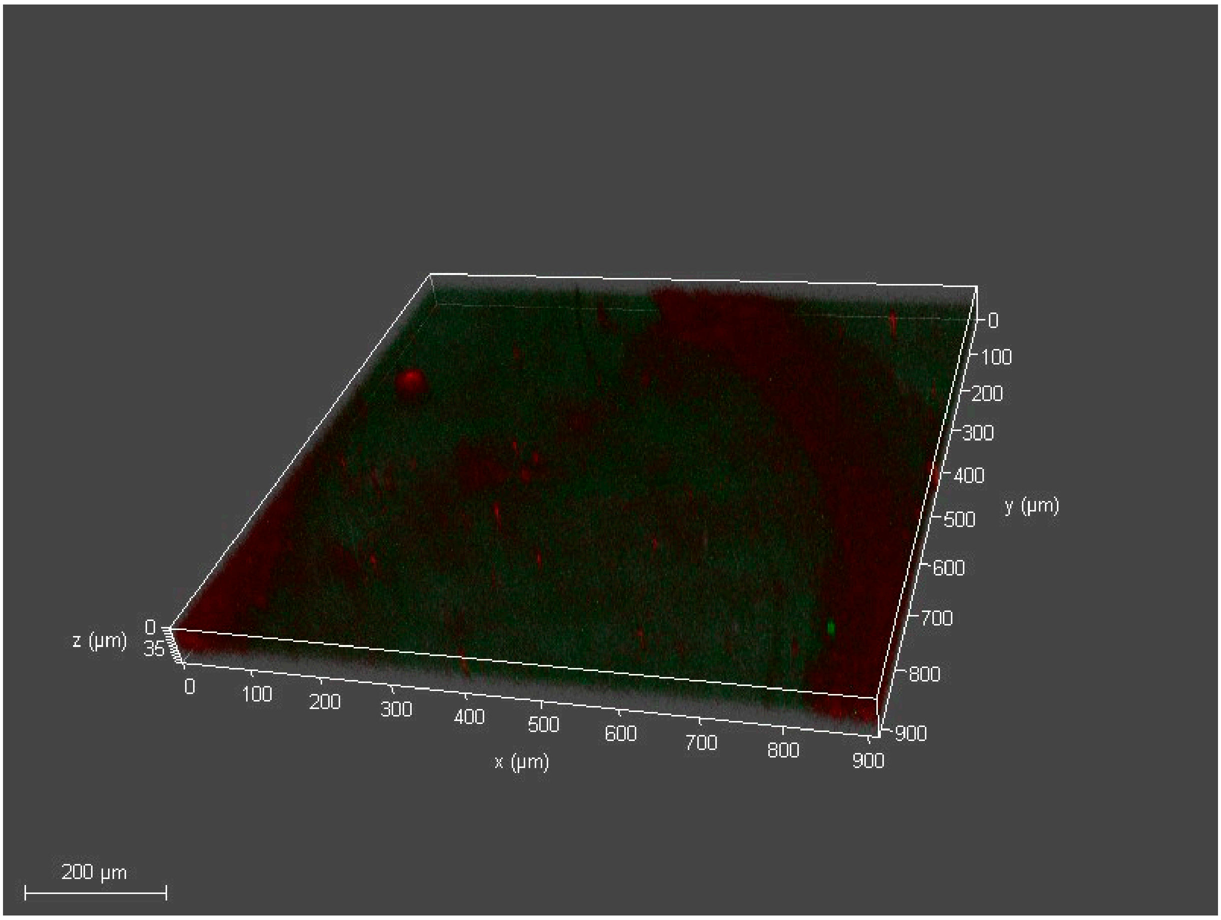

B. Scanning Electron Microscopy (SEM) Raw Images

1- SEM (control)

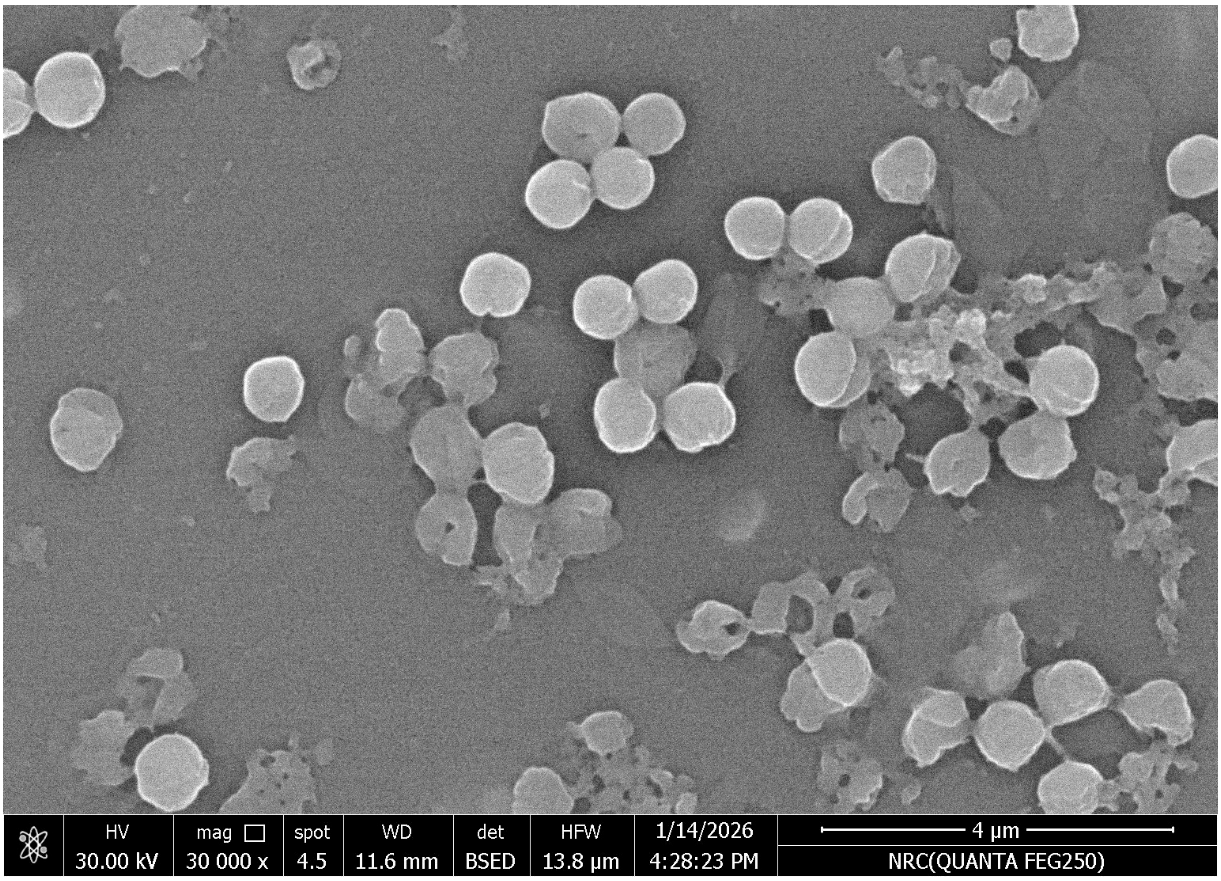

2-SEM (THY)

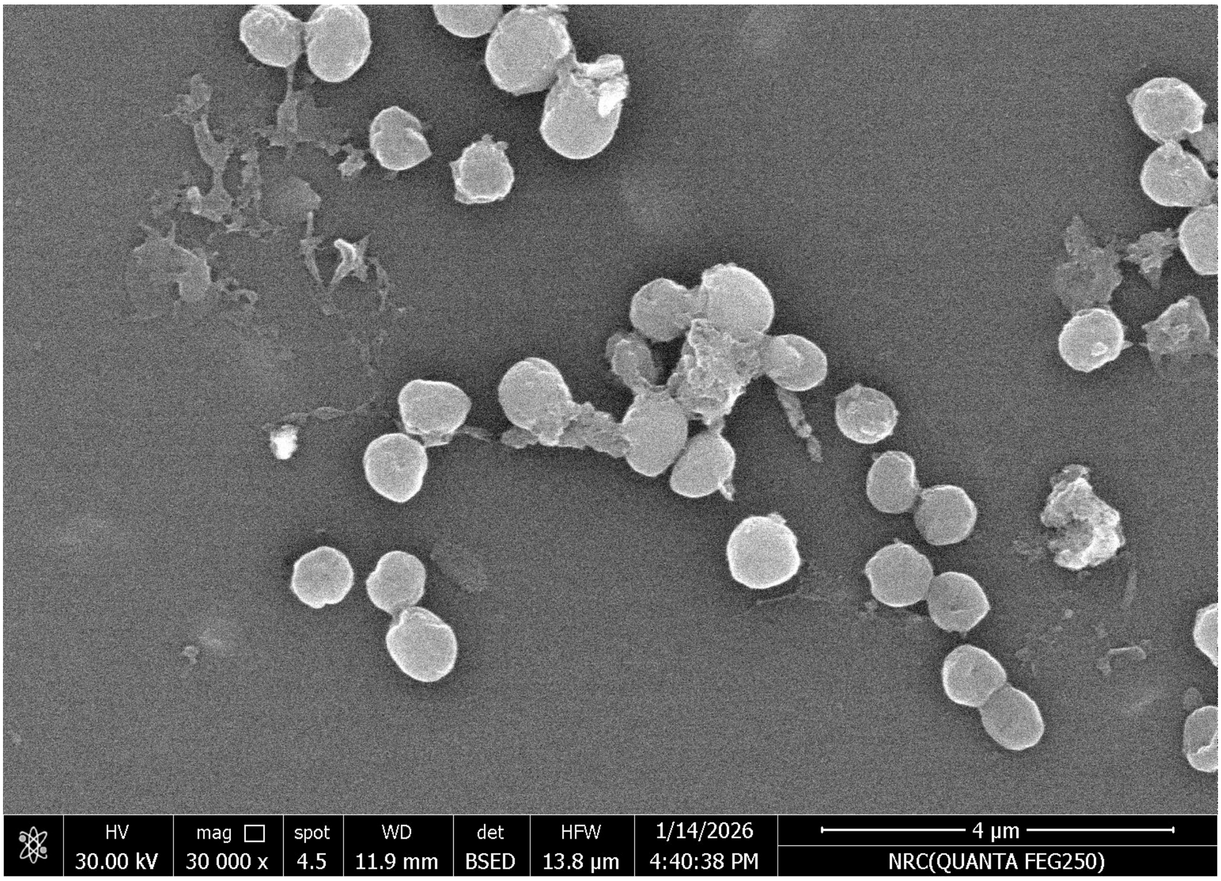
HV
30.00 kV
mag
30 000 x
spot
4.5
WD
11.9 mm
det
BSED
HFW
13.8 µm
1/14/2026
4:40:38 PM
4 µm
NRC(QUANTA FEG250)

3-SEM (THY-LPX)

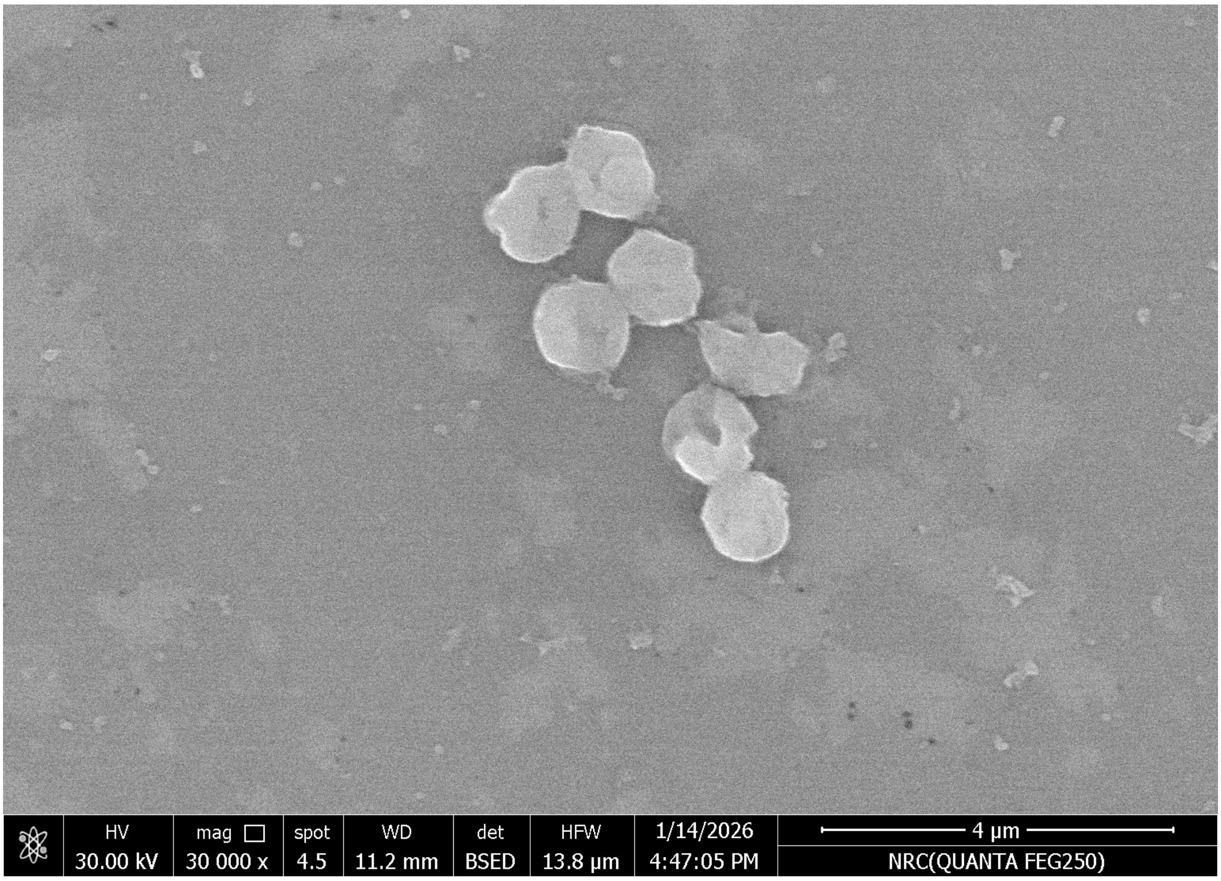
HV
30.00 kV
mag
30 000 x
spot
4.5
WD
11.2 mm
det
BSED
HFW
13.8 µm
1/14/2026
4:47:05 PM
4 µm
NRC(QUANTA FEG250)
